# Amyloid‐Induced Network Resilience and Collapse in Alzheimer's Disease: Insights From Computational Modeling

**DOI:** 10.1111/ejn.70713

**Published:** 2026-10-20

**Authors:** Ediline L. F. Nguessap, Damien Depannemaecker, Fernando F. Ferreira

**Affiliations:** ^1^ Department of Physics FFCLRP/University of São Paulo São Paulo Brazil; ^2^ Aix Marseille Université Institut National de la Santé et de la Recherche Médicale, Institut de Neurosciences des Systèmes (INS) UMR1106 Marseille France

**Keywords:** Alzheimer's disease, amyloid proteins, resilience, spikes‐firing rate, synchronization

## Abstract

Alzheimer's disease is marked by progressive synaptic loss and cognitive decline, with amyloid‐beta accumulation playing a central pathological role. Although the molecular effects of amyloid‐beta are well‐characterized, the cascade from local synaptic dysfunction to large‐scale network collapse remains poorly understood. We present a biophysically grounded computational model that integrates small‐world network topology, Izhikevich neuron dynamics, short‐term plasticity, and amyloid‐beta‐induced synaptic pruning to investigate how amyloid burden degrades functional brain connectivity over time. Crucially, we simulate the evolving network dynamics across disease stages, revealing how firing rate, synchrony, variability, and Fano factor evolve in response to progressive structural degradation. Our results demonstrate a two‐phase deterioration: Initial compensatory dynamics followed by abrupt disintegration of coordinated activity. This temporal dissociation mirrors clinical observations and offers mechanistic insight into Alzheimer's disease progression. We further identify a critical accumulated amyloid dose beyond which network collapse becomes inevitable, preceded by early‐warning indicators such as declining global efficiency and the spectral radius of the network connections. Network topology, synaptic time constants, and repair mechanisms strongly influence resilience, with small‐world networks showing delayed collapse and greater functional compensation. Finite‐size scaling reveals that larger networks experience earlier and sharper phase transitions. Ultimately, by establishing a mathematical link between microscopic synaptic degradation and macroscopic network failure, our findings suggest that if experimental proxies for the spectral radius can be identified, they would serve as highly sensitive early‐warning indicators of impending network collapse. This theoretical framework offers new mechanistic targets for the early detection and therapeutic monitoring of neurodegenerative diseases.

## Graphical abstract

We present a multiscale computational model linking amyloid‐beta accumulation to brain network collapse in Alzheimer's disease. The spectral radius serves as an early‐warning biomarker, anticipating structural collapse by ~47 months. Progressive synaptic degradation drives a transition from synchronized activity through functional subpopulations to global silence, with gamma‐band oscillations persisting (albeit with reduced power) until late‐stage failure.

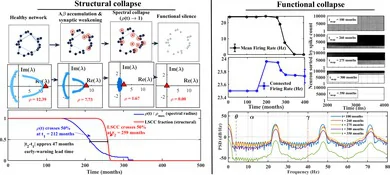

**Abbreviations**
ad
: Alzheimer's disease
Aβ: amyloid‐beta
CL: amyloid concentration level
CV: coefficient of variation
GE: global efficiency
LSCC: largest strongly connected component
PSD: power spectral density
STP: short‐term plasticity

## 1 Introduction

Alzheimer's disease (ad) is the most common form of dementia and is characterized by progressive cognitive decline, synaptic loss, and neuronal death. A hallmark of ad pathology is the accumulation of amyloid‐beta Aβ plaques, which disrupt synaptic function and trigger a cascade of neurodegenerative Aβ processes (Villemagne et al. 2013; Selkoe and Hardy 2016). Despite extensive experimental studies, the link between molecular‐scale accumulation and large‐scale network collapse remains poorly understood. Clinical observations suggest that functional brain networks exhibit altered connectivity and reduced efficiency long before the appearance of overt structural damage or cognitive symptoms (Sperling et al. 2011; Jones et al. 2016).

Computational modeling has emerged as a powerful approach to explore the mechanisms of disease progression across spatial and temporal scales. Previous work has shown that Alzheimer's disease‐related damage propagates nonrandomly across the brain, preferentially targeting hub regions and disrupting integration (Raj et al. 2012; Kashyap et al. 2019). Other studies have explored the impact of excitability, inflammation, or neurovascular coupling on network vulnerability (de Haan et al. 2017; Gu et al. 2021), and some have incorporated spreading models of pathology over structural connectomes. However, many of these models focus solely on structural disconnection or static analysis, and few address how synaptic failure translates into dynamic alterations in spiking activity.

Moreover, experimental evidence indicates that synaptic plasticity and compensatory sprouting are active during the early stages of Alzheimer's disease (Zhang et al. 2023), suggesting that the brain attempts to counteract degradation before reaching a critical threshold. Understanding how these compensatory mechanisms interact with network architecture and neuronal dynamics is essential for identifying early biomarkers and intervention windows.

Although prior studies have modeled amyloid‐driven synaptic degradation using structural networks (Raj et al. 2012; Kashyap et al. 2019), they often omit dynamic spiking activity, synaptic plasticity, or repair mechanisms, limiting their ability to capture the full trajectory of Alzheimer's progression. Other models have addressed hyperexcitability (de Haan et al. 2017) or propagation dynamics (Gu et al. 2021) but typically neglect functional metrics or do not incorporate biologically grounded thresholds for collapse. Moreover, few frameworks integrate both network topology and intrinsic neuronal excitability when evaluating resilience. Additionally, many existing models rely on rate‐based or abstracted node dynamics, which are less suited to capturing temporal patterns such as spike bursts and firing variability which are features increasingly linked to early Alzheimer's disease signatures in electrophysiological data (Palop and Mucke 2016). A spiking‐neuron–based framework is therefore essential for capturing dynamic signatures of collapse and compensatory coordination.

In contrast, our work presents a unified modeling framework that incorporates small‐world topology, Izhikevich neuronal dynamics, short‐term synaptic plasticity, and Aβ‐induced synaptic pruning, along with activity‐dependent repair. Short‐term plasticity (STP), in particular, modulates synaptic efficacy on the timescale of milliseconds to seconds, shaping the propagation of activity in networks undergoing stress. Its role in early compensation has been observed experimentally, but is rarely incorporated into computational Alzheimer's disease models. This enables us to capture both structural degradation and emergent functional collapse, characterize early‐warning signals, and explore how both synaptic timescales and neuronal excitability influence the temporal dynamics of network failure.

In the following sections, we develop and analyze this biophysically motivated network model to simulate the progressive effects of Aβ toxicity on neural connectivity and dynamics. We identify critical thresholds, quantify early indicators of collapse, and assess how network topology, plasticity, and intrinsic dynamics shape the trajectory of disease progression.

## 2 Models

We exploit the separation of timescales between (i) the month‐to‐yearlong degradation of network structure due to amyloid accumulation and (ii) the millisecond‐scale spiking dynamics of neurons embedded in these networks. We first model the progressive structural degradation of synaptic connectivity driven by Aβ accumulation, characterizing it using graph‐theoretic metrics (largest strongly connected component [LSCC], global efficiency [GE], and susceptibility [*V*]). We demonstrate a critical transition in network connectivity at a well‐defined amyloid burden. We then study the dynamics of spiking neurons (Izhikevich model with STP) on static network snapshots extracted at key disease stages: precollapse, collapse‐onset, and postcollapse, treating connectivity as fixed on the millisecond timescale.

### 2.1 Network Construction

The neuronal network was implemented as a directed Watts–Strogatz small‐world network with *N* = 10^4^ neurons and average degree *k* = 20, parameters chosen to match the connectivity statistics observed in mammalian cortical microcircuits (Markram et al. 2015). All synaptic weights were initialized to *W*_*0*_ = 1, representing normalized connection strengths. This uniform initialization allows clear observation of amyloid‐induced degradation effects without confounding by initial weight heterogeneity.

Synapses (edges) are removed from the network when their weight falls below *W*_*min*_ *= W*_*0*_ */(2e)*. This threshold is derived from the minimum functional connectivity required for signal propagation in spiking network models (Vogels et al. 2011). It models synapse elimination when postsynaptic potentials drop below 18% of their initial strength, consistent with electrophysiological measurements in ad pathology. Neurons that lose all incoming connections become functionally isolated and quiescent but are retained in the graph. This ensures that the total number of nodes remains constant at *N* = 10^4^ (unless otherwise stated) throughout the simulation. By using this fixed initial ***N*** as a stable reference denominator, topological metrics (e.g., LSCC fraction and GE) accurately reflect the proportion of the original network that remains functional at each time point (DeKosky and Scheff 1990).

### 2.2 Amyloid Dynamics

Amyloid accumulation followed logistic growth with microglial clearance:

(1) $$\begin{array}{c} \frac{\mathrm{dA}}{\mathrm{dt}}=\underset{\overbrace{\text{Autocatalytic growth}}}{k_{A}A\left(1-\frac{A}{A_{\max}}\right)}-\underset{\overbrace{\text{Clearance}}}{\mathrm{\gamma A}} \end{array}$$

This logistic formulation captures the empirically observed sigmoidal progression of Aβ plaque accumulation documented in longitudinal positron emission tomography cohorts (DeKosky and Scheff 1990; Villemagne et al. 2013). The autocatalytic growth term mathematically represents the self‐seeding kinetics of Aβ fibril formation and nucleation, bounded by the physical saturation of available aggregation sites. To ensure translational validity, the growth rate *k*_*A*_ = 0.03 month^−1^ and the theoretical carrying capacity *A*_*max*_ = 150 CL were strictly calibrated against clinical data to replicate the characteristic 15–20 years preclinical incubation window observed in patient cohorts (Jack et al. 2013). Conversely, the clearance term *γ·A* employs a decay rate of *γ* = 0.005 month^−1^ to simulate continuous microglial phagocytosis and physiological clearance mechanisms at empirically verified rates (Yuan et al. 2023).

When amyloid levels exceeded the toxicity threshold *A*_*th*_ = 25 CL, which matches positron emission tomography and defined as amyloid positivity (Klunk et al. 2022; Jagust and Landau 2021), synaptic weights decayed exponentially as *W(t) = W₀·exp(−t/τ_s_)*; with pruning probability:

(2) $$\begin{array}{c} P_{\text{prune}}=1-\exp\left(-\lambda \cdot \left(A-A_{\mathrm{th}}\right)\right) \end{array}$$

The toxicity factor *λ* = 0.001 CL^−1^ was derived from postmortem studies correlating amyloid load with synapse loss (DeKosky and Scheff 1990; Terry et al. 1991). The synaptic decay time constants *τ*_*s*_ ∈ {60, 200} months were derived from two complementary sources: (i) longitudinal clinical studies showing that the interval between amyloid positivity and cognitive decline spans approximately 5–17 years (Jack et al. 2013; Burgold et al. 2014), and (ii) postmortem histological quantification of synapse loss rates in ad (DeKosky and Scheff 1990; Terry et al. 1991).

Crucially, the amyloid burden *A(t)* enters the model as a damage rate rather than an instantaneous state variable. At each monthly time step, an existing synapse is removed with probability *P*_*prune*_, so it survives that step with probability *exp[−λ(A − A*_*th*_ *)]*. Because a synapse present at time *t* must have escaped pruning at every preceding step because the onset of toxicity, and assuming independent survival probabilities, the cumulative surviving‐edge fraction is the product of these individual probabilities. Taking the continuum limit, we define the accumulated suprathreshold amyloid dose:

(3) $$\begin{array}{c} D\left(t\right)=\int_{\;t\prime }^{t}\left[A\left(u\right)-A_{\mathrm{th}}\right]\mathrm{du}, \end{array}$$

where *t′* is the time after which a pruned synapse's weight actually falls below the removal threshold *W*_*min*_ and is physically deleted from the network. The surviving‐edge fraction is then given by: *p(t) = exp[−λD(t)]*.

This formulation reveals that network degradation depends not only on how far the amyloid burden exceeds the toxicity threshold *A*_*th*_, but critically on how long the network is exposed to these suprathreshold levels. The percolation of the network is therefore governed by the accumulated dose *D(t)* reaching a critical value *D*_*c*_, rather than by the instantaneous amyloid level *A(t)*. This implies that the specific amyloid concentration at which collapse is observed depends on the rate of amyloid accumulation and the synaptic decay timescale, rather than being a universal constant of the disease.

The structural evolution is simulated in discrete monthly time steps (*Δt* = 1 month) following a strict sequence: (i) The amyloid level A is updated according to Equation (1); (ii) every existing synapse in the network is independently tested for pruning with probability P_prune; if selected, the synaptic weight is instantaneously reset to *W(t) = W₀·exp(−t/τ*_*s*_ *)*, representing the cumulative effect of amyloid toxicity at that disease stage, and the synapse is immediately removed if its weight falls below *W*_*min*_ *= W₀/(2e)*; and (iii) if *A < A*_*th*_, repair and axonal sprouting mechanisms are applied to the surviving synapses. Thus, pruning is performed on a synapse‐by‐synapse basis at every monthly time step rather than through a simultaneous global weakening of all connections. This procedure produces a gradual network degradation that is more consistent with the progressive nature of neurodegeneration.

### 2.3 Repair and Reconnection Mechanism

The model incorporates two biologically observed compensation mechanisms: synaptic repair and axonal sprouting. Existing synapses repair with probability:

(4) $$\begin{array}{c} p_{\text{repair}}=\frac{0.02}{\sqrt{N}}\left(1-e^{-0.01\left(A_{\mathrm{th}}-A\right)}\right) \end{array}$$

Each existing synapse (*i, j*) is strengthened with probability *P*_*repair*_ as

(5) $$\begin{array}{c} w_{\mathrm{ij}}\leftarrow \min\left(W_{0},w_{\mathrm{ij}}\cdot \left(1+\alpha \right)\right) \end{array}$$

*α* = 3% is the repair strength factor matching measured long‐term potentiation magnitudes in early ad (Selkoe and Hardy 2016). New connections form at half the repair rate probability (*P*_*repair*_/2) with initial weight 0.5*W*_*0*_, simulating axonal sprouting (DeKosky and Scheff 1990).

### 2.4 Graph Metrics

We compute the following graph metrics to quantify network integrity:

#### 2.4.1 LSCC

The LSCC is the largest subset of neurons in which every neuron is reachable from every other neuron in the subset via directed paths. The size of the LSCC is normalized by the total number of neurons: LSCC *= |Largest* SCC*|/N* where |Largest SCC| is the number of neurons in the LSCC.

#### 2.4.2 GE

GE measures the efficiency of information transfer across the network. It is defined as the average inverse shortest path length between all pairs of neurons:

(6) $$\begin{array}{c} E_{\text{global}}=\frac{1}{N\left(N-1\right)}\sum_{i\neq j}\frac{1}{d_{\mathrm{ij}}} \end{array}$$

where *N*: Total number of neurons and *d*_*ij*_: Shortest path length between neurons *i* and *j*.

#### 2.4.3 Susceptibility of the LSCC/Fluctuation of the Mean of the LSCC

To identify critical points in the network, we calculate the susceptibility *V* of the LSCC, which measures the fluctuations of the LSCC in relation to its average value in several independent realizations of the network evolution. The susceptibility is defined as

(7) $$\begin{array}{c} V=\frac{\left\langle {\left(\text{LSCC}\right)}^{2}\right\rangle }{\left\langle \text{LSCC}\right\rangle }-\left\langle \text{LSCC}\right\rangle , \end{array}$$

where ⟨*LSCC*⟩ is the average of the LSCC in *n*_real_ activities:

(8) $$\begin{array}{c} \left\langle \text{LSCC}\right\rangle =\frac{1}{n_{\text{real}}}\sum_{r=1}^{n_{\text{real}}}{\text{LSCC}}_{r} \end{array}$$

and ⟨*(LSCC)* ^*2*^⟩ is the mean square of the LSCC *n*_real_ in realizations:

(9) $$\begin{array}{c} \left\langle {\left(\text{LSCC}\right)}^{2}\right\rangle =\frac{1}{n_{\text{real}}}\sum_{r=1}^{n_{\text{real}}}{\left({\text{LSCC}}_{r}\right)}^{2} \end{array}$$

with LSCC_*r*_ is the value of LSCC the *r‐th* realization.

This susceptibility measure evolves dynamically, identifying time‐dependent critical points in the network. Because the amyloid burden *A(t)* acts as a strictly monotonic exogenous driver, the critical time of collapse is mathematically equivalent to a critical amyloid threshold. However, because the topological collapse is a cumulative stochastic process (pruning vs. repair) rather than an instantaneous phase transition, we primarily track the critical time *T*_*c*_, while explicitly reporting the corresponding amyloid burden *A (T*_*c*_ *)* at which the connectivity collapses. Fluctuations in *V(t)* reflect temporal instability in LSCC size, signaling progressive network deterioration or phase transitions as the system approaches collapse.

### 2.5 Neuronal Dynamics With STP

We extracted adjacency matrices at critical stages: (1) precollapse (healthy state), (2) collapse onset (amyloid threshold), and (3) postcollapse (disintegrated state). Neuronal dynamics were simulated on each static snapshot using the Izhikevich model to isolate the impact of connectivity changes on spiking activity. Although this assumes synaptic weights remain fixed during simulation windows, this quasistatic approximation is justified by the timescale separation between amyloid accumulation in months and years (Jack et al. 2013; Burgold et al. 2014) and spiking dynamics in milliseconds (Markram et al. 1998; Izhikevich 2003; Markram et al. 2015). This timescale separation is supported by computational studies of ad (Stam et al. 2007; Dehghani et al. 2016). To study the impact of synaptic dynamics on neuronal activity, we simulate the Izhikevich neuron model coupled with STP. The neuronal and synaptic dynamics are governed by the following equations:

#### 2.5.1 Neuron Dynamics

The membrane potential *v*_*i*_ and recovery variable *u*_*i*_ for each neuron evolve according to the Izhikevich model (Izhikevich 2003):

(10) $$\frac{dv_{i}}{\mathrm{dt}}=0.04v_{i}^{2}+5v_{i}+140-u_{i}+I+I_{i}^{\mathrm{syn}}$$

(11) $$\begin{array}{c} \frac{du_{i}}{\mathrm{dt}}=a\left(bv_{i}-u_{i}\right) \end{array}$$

with spike resetting:

(12) $$\text{if}\;v_{i}\geq 30\mathrm{mV},\left\{\begin{array}{c} v_{i}\leftarrow c\\ u_{i}\leftarrow u_{i}+d \end{array}\right.$$

We explore variations in intrinsic excitability by adjusting the parameters *a* and *d*, which modulate neuronal recovery and after‐spike reset, respectively.

#### 2.5.2 STP Dynamics and Input Current

To align with standard matrix notation, we explicitly define *j* as the presynaptic neuron and *i* as the postsynaptic neuron. Short‐term synaptic plasticity is implemented as a dynamic modulation of synaptic efficacy for each synapse (*j* → *i*). For each synapse *x*_*ij*_ *(t)*, we define the facilitation (use of synaptic efficacy); *R*_*ij*_ *(t)*: the fraction of synaptic resources available; *U*: baseline utilization; *τ*_*facil*_ and *τ*_*rec*_ the time constants for facilitation and recovery (Markram et al. 1998; Zucker and Regehr 2002; Markram et al. 2015).

After a spike from neuron:

(13) $$\begin{array}{c} x_{\mathrm{ij}}\leftarrow x_{\mathrm{ij}}+U\left(1-x_{\mathrm{ij}}\right);R_{\mathrm{ij}}\leftarrow R_{\mathrm{ij}}\cdot \left(1-x_{\mathrm{ij}}\right) \end{array}$$

Between spikes, the variables evolve as

(14) $$\begin{array}{c} \frac{dx_{\mathrm{ij}}}{\mathrm{dt}}=\frac{U-x_{\mathrm{ij}}}{\tau_{\text{facil}}};\frac{dR_{\mathrm{ij}}}{\mathrm{dt}}=\frac{1-R_{\mathrm{ij}}}{\tau_{\mathrm{rec}}} \end{array}$$

The effective synaptic weight becomes

(15) $$\begin{array}{c} w_{\mathrm{ij}}^{\mathrm{eff}}\left(t\right)=w_{\mathrm{ij}}\cdot x_{\mathrm{ij}}\left(t\right)\cdot R_{\mathrm{ij}}\left(t\right) \end{array}$$

And the synaptic input to neuron (*j* → *i*) is

(16) $$\begin{array}{c} I_{i}^{\mathrm{syn}}\left(t\right)=\sum_{j\in \mathrm{pre}\left(i\right)}w_{\mathrm{ij}}^{\mathrm{eff}}\left(t\right)\cdot \delta_{j}\left(t\right) \end{array}$$

where *δ*_*j*_ *(t) = Σ*_*k*_ *δ(t − t*_*j*_^*(k)*^ *)* represents the spike train of the presynaptic neuron *j*, and *t*_*j*_^*(k)*^ denotes the precise time of the *k‐th* spike emitted by that neuron. In our discrete‐time numerical implementation *(Δt = 1 ms)*, this continuous formulation is realized via an event‐driven update rule: Whenever a presynaptic spike occurs, the effective weight *w*_*ij*_^*eff*^ is added instantaneously to the postsynaptic membrane potential *v*_*i*_, exactly preserving the continuous‐time magnitude without requiring scaling by the integration time step.

Population synchrony reported in Figure 11 Panel (b) is quantified as the temporal standard deviation of the instantaneous population firing rate. Let *R(t)* represent the total number of spikes occurring at time *t* across the set of connected neurons. The population synchrony is then defined as *σ[R(t)],* where *σ* denotes the standard deviation across the analysis window. This metric serves as a robust proxy for network‐wide coordination: Elevated temporal fluctuations indicate synchronized population bursts (where many neurons fire simultaneously), whereas reduced fluctuations reflect asynchronous or disorganized firing patterns.

In our computational framework, the implementation of repair and reconnection mechanisms is biologically motivated and temporally aligned with amyloid‐beta progression expressed in Centiloid (CL) units. Research suggests that Aβ begins to accumulate silently during the preclinical phase of Alzheimer's disease, with neurotoxic effects becoming detectable beyond 25 CL, which is the threshold for Aβ‐positivity (Jagust and Landau 2021). Our model assumes that neuronal dysfunction may begin subtly in this early phase, prompting compensatory repair mechanisms such as axonal sprouting, synaptic reformation, or homeostatic plasticity (Tampellini 2015; Selkoe and Hardy 2016). Experimental data suggest that once Aβ levels surpass the threshold and continue to rise, especially as they approach 75 CL (associated with cognitive impairment), the efficiency of these endogenous repair mechanisms diminishes (Villemagne et al. 2013; Jack et al. 2018). However, our model incorporates pre‐symptomatic repair mechanisms that become inactive when amyloid levels *A* reach the positivity threshold (*A* ≥ 25 CL).

The network equations are solved numerically using the fourth‐order Runge–Kutta method to ensure accurate integration of neuronal and synaptic dynamics with time step of 1 ms. Unless otherwise noted, the simulations use the following fixed parameter: *λ* = 0.001, A_th_ = 25 CL *τ*_*s*_ = 120 months, *N =* 10^4^, *A*_*max*_ = 150 CL*, γ =* 0.005 month^−1^, *k*_*A*_ *= 0.02*.

## 3 Structural Degradation and Network Resilience

### 3.1 Network Degradation: LSCC Collapse and Critical Threshold

The constructed network is of average clustering coefficient of ≈0.5, and the shortest path length of ≈4.4. Figure 1 provides a comprehensive illustration of the temporal evolution of Alzheimer's pathology and its impact on network connectivity and compensatory mechanisms. Panel (a) shows the sigmoidal progression of amyloid‐beta accumulation, modeled through logistic growth dynamics with three phases: slow buildup (0–150 months, *R* ^*2*^ = 0.98), rapid expansion (150–300 months), and saturation (*t >* 300 months). Two key thresholds are marked: the toxicity threshold at 25 CL (dash red), where synaptic degradation begins, and collapses abruptly once the accumulated amyloid dose reaches the critical threshold (which corresponds to *~*75 CL (solid red) under baseline parameters), corresponding to irreversible network failure. This growth pattern mirrors clinical observations, highlighting a long silent phase, a rapid expansion stage, and eventual saturation.

**FIGURE 1:**
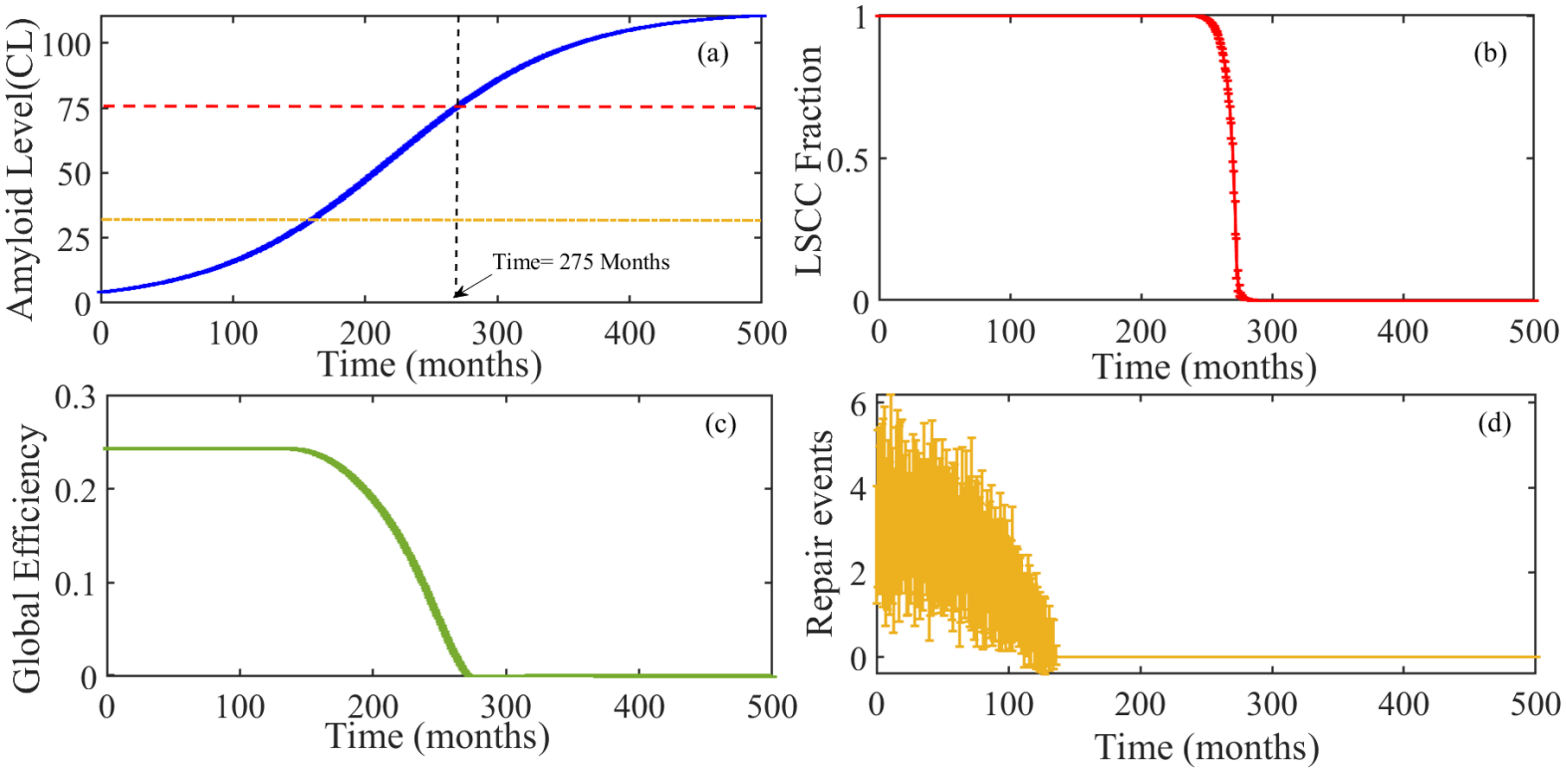
Network degradation under progressive amyloid‐beta accumulation: (a) Aβ accumulation over time showing two key thresholds: 25 CL (toxicity onset) and ~75 CL (collapse for baseline parameters). (b) Fraction of the largest strongly connected component (LSCC) collapses after 75 CL. (c) Global efficiency (GE) declines earlier, indicating functional degradation before structural collapse. (d) Repair events peak early and cease once exceeds 25 CL Parameters: *λ* = 0.01,*τ*_*s*_ = 120 months, *N* = 10^4^. Error bands: SEM (*n* = 20).

Panel (b) captures the fraction of the LSCC, a structural marker of global network integration. The LSCC remains largely intact during early amyloid buildup but collapses abruptly once Aβ surpasses the 75 CL threshold. This sharp, nonlinear decline indicates a critical transition resembling a phase shift, where resilience gives way to fragmentation.

In contrast, Panel (c) shows that GE, a measure of functional integration, begins to decline significantly before LSCC breakdown. This early decline acts as a leading indicator of structural collapse, consistent with the theory of critical slowing down, and suggests that functional disconnection may serve as a sensitive biomarker of preclinical Alzheimer's progression.

Finally, Panel (d) illustrates the trajectory of repair events, such as synaptic reinforcement and axonal sprouting. These compensatory processes peak just below the 25 CL threshold and cease rapidly thereafter, indicating a narrow window of effective plasticity during early amyloid accumulation. This dynamic justifies the model's assumption that repair mechanisms operate only below the toxicity threshold.

Collectively, the figure encapsulates the multiphase degradation of brain networks in Alzheimer's disease, linking molecular pathology to the functional and structural collapse of neuronal connectivity.

### 3.2 Effects of Network Size and Finite‐Size Scaling

The structural vulnerability of neuronal networks under amyloid stress varies and depends on the network size. To further investigate it, we systematically analyzed the effect of network size on the timing and nature of connectivity collapse. Figure 2 examines how network size influences the progression and abruptness of connectivity loss under amyloid‐induced degeneration, revealing a counterintuitive finding: Larger networks exhibit earlier and more abrupt collapse. Panel (a) displays the evolution of the fraction of the LSCC over time for different network sizes (100 to 10,000). Contrary to expectations that increased size might enhance resilience, the results show that LSCC fragmentation occurs sooner in larger networks.

**FIGURE 2:**
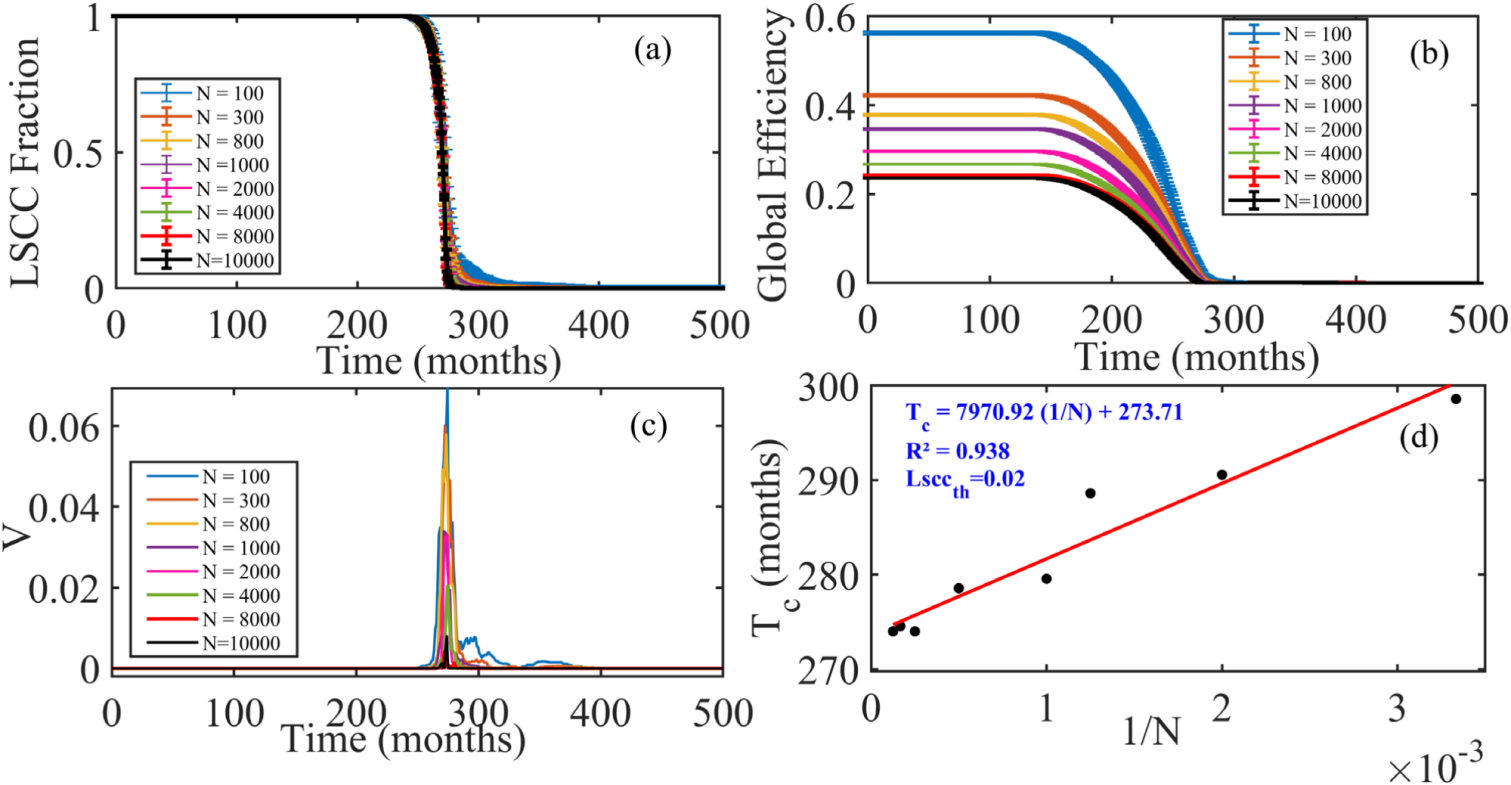
Network size modulates collapse timing and sharpness: (a) LSCC fraction over time for networks with *N* = 100 to 10,000. Larger networks collapse earlier and more abruptly. (b) GE declines sooner in larger networks. (c) LSCC susceptibility *V* peaks near the collapse point and sharpens with increasing *N*. (d) Collapse time *T*_*c*_ scales inversely with *N*, indicating a finite‐size transition. Error bands: SEM (*n* = 20).

The inset highlights that after collapse, the LSCC remains below 0.1 for larger networks, indicating a sharper and more permanent breakdown of global connectivity. Panel (b) confirms this vulnerability by showing GE as a function of time: networks with more neurons suffer earlier declines in functional efficiency. This result implies that although larger networks have higher baseline integration, they are also more sensitive to perturbations once degradation begins.

Panel (c) introduces the susceptibility *V* of the LSCC, which measures fluctuations across realizations and peaks near the transition point for all network sizes. As *N* increases, this peak becomes sharper and shifts leftward, consistent with the hallmark of a second‐order phase transition and supporting the notion of critical slowing down in Alzheimer's disease progression (Scheffer et al. 2012). Finally, Panel (d) presents a finite‐size scaling analysis, plotting the critical collapse time *T*_*c*_ (defined as the time at which LSCC drops below 0.02) against *1/N*. The linear relationship observed (*R* ^*2*^ = 0.938) suggests that the critical point becomes increasingly well‐defined as the system size grows, aligning with finite‐size scaling theory in statistical physics.

Together, these findings indicate that larger neuronal networks, despite their higher initial robustness, are more prone to sudden and earlier transitions into dysfunctional states under amyloid stress. This aligns with prior computational work by Kashyap et al. (2019), which reported more abrupt transitions in large‐scale systems due to amplified sensitivity to structural perturbations. These results underscore the importance of accounting for network size in models of neurodegeneration and suggest that individual variability in connectome scale may partly explain differing disease trajectories in Alzheimer's patients.

### 3.3 Effects of Synaptic Time Constant (*τ*_*s*_)

The differential effects of *τ*_*s*_ values reveal a fundamental trade‐off between synaptic resilience and network stability in Alzheimer's disease progression. Shorter synaptic time constants (*τ*_*s*_) accelerate network degradation, whereas longer *τ*_*s*_ delay the collapse of the LSCC.

To explore how the temporal properties of synaptic weakening influence network resilience, we analyzed the effect of varying the synaptic decay time constant *τ*_*s*_ on network degradation dynamics. Figure 3 presents the evolution of both the LSCC and GE for networks subjected to different values of *τ*_*s*_. These simulations reveal a critical trade‐off between synaptic vulnerability and the timing of network collapse. In Panel (a), networks with shorter decay constants (e.g., *τ*_*s*_ *=* 60 months) exhibit early and rapid LSCC collapse, indicating accelerated structural degradation. This dynamic corresponds to more aggressive forms of Alzheimer's disease, where synaptic integrity deteriorates swiftly and irreversibly (Arendt 2009).

**FIGURE 3:**
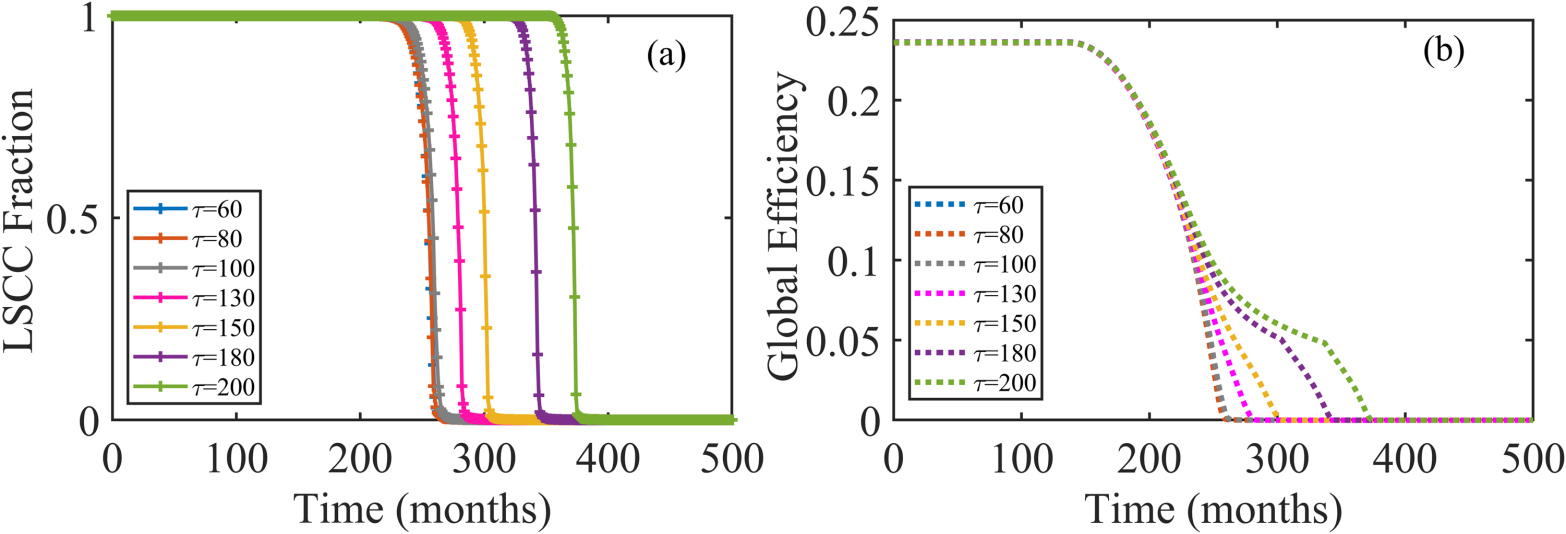
Effect of synaptic decay time *τ*_*s*_ on network resilience: (a) LSCC trajectories for different *τ*_*s*_. Larger *τ*_*s*_ delays collapse. (b) GE declines earlier for shorter *τ*_*s*_. Networks with slower synaptic decay retain functional connectivity longer.

Conversely, longer decay constants (e.g., *τ*_*s*_ = 200 months) delay the collapse, preserving the LSCC even as amyloid levels rise. This resilience reflects a compensatory phase in which network topology buffers against toxicity; an effect analogous to the cognitive reserve observed in some ad patients (Stam et al. 2007). Panel (b) further illustrates that GE deteriorates significantly earlier in fast‐decay scenarios, emphasizing that functional disintegration precedes structural breakdown. Notably, the time lag between amyloid crossing the toxicity threshold (*A*_*th*_ = 25 CL) and LSCC failure increases with *τ*_*s*_, indicating a biphasic progression: an initial period of subclinical compensation, followed by an abrupt transition to system‐wide failure.

This dissociation of functional and structural collapse highlights the importance of *τ*_*s*_ as a tunable parameter that captures individual differences in disease trajectories. Moreover, the results suggest that patients with longer synaptic decay times may benefit more from early therapeutic interventions aimed at preserving synaptic efficacy. These findings are consistent with clinical studies showing that synaptic biomarkers decline years before cognitive symptoms appear (Jones et al. 2016; Chia et al. 2024). By operationalizing *τ*_*s*_ within a dynamic framework, our model links microscale degeneration to macroscale network failure, providing mechanistic insight into how subtle changes in synaptic resilience modulate Alzheimer's progression.

To further quantify the impact of synaptic decay dynamics on network resilience, we investigated the relationship between the synaptic time constant *τ*_*s*_ and the critical collapse time *T*_*c*_, defined as the time at which the LSCC drops below 0.02. This analysis is presented in Figure 4, which shows a clear monotonic relationship: As *τ*_*s*_ increases, the value of *T*_*c*_ is systematically delayed. This trend demonstrates that longer lasting. Synapses significantly extend the network's ability to resist collapse under amyloid stress.

**FIGURE 4:**
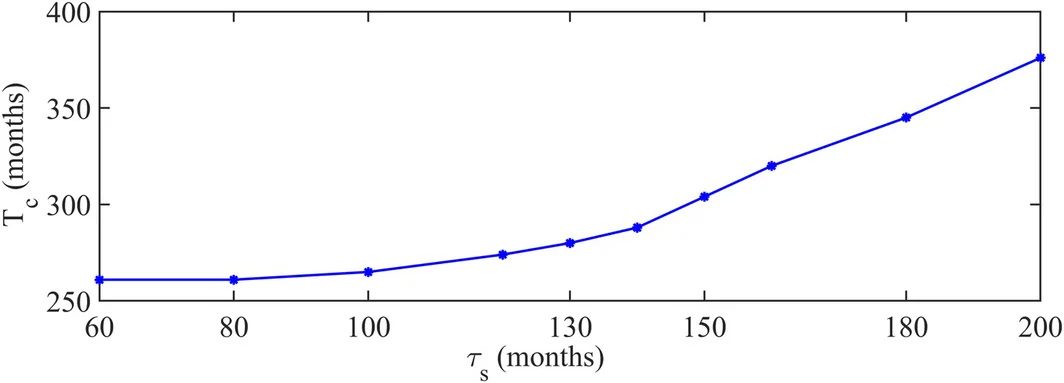
Critical collapse time increases with synaptic decay time: Relationship between *τ*_*s*_ and the critical collapse time *T*_*c*_. Longer lasting synapses delay network failure, supporting the role of *τ*_*s*_ in compensatory resilience.

The approximately linear increase in *T*_*c*_ with *τ*_*s*_ supports the hypothesis that synaptic longevity buffers the network against cumulative damage, allowing for a prolonged phase of functional compensation. This result not only reinforces the biphasic degradation trajectory observed in Figure 3 but also suggests that *τ*_*s*_ serves as a key parameter in modulating the onset of catastrophic network failure. The clinical implication is that interindividual differences in synaptic decay dynamics may partially account for the observed variability in disease progression rates among Alzheimer's disease patients (Stern 2012; Villemagne et al. 2013).

From a theoretical standpoint, the nearly linear relationship between *τ*_*s*_ and *T*_*c*_ aligns with the idea of delayed transitions in complex systems, where slower decay rates permit extended stability until a critical threshold is breached (Scheffer et al. 2012). This finding also highlights the potential of *τ*_*s*_ as a surrogate biomarker or a target for therapeutic modulation, suggesting that interventions aimed at slowing synaptic decay could meaningfully postpone the functional collapse of large‐scale brain networks.

### 3.4 Effects of Rewiring Probability (p_rewire_)

To investigate how network topology influences vulnerability to amyloid‐induced collapse, we systematically varied the rewiring probability p_*rewire*_ in the Watts–Strogatz model, transitioning from ordered lattices (p_rewire_ = 0) to small‐world (p_rewire_ = 0.1,0.5) and fully random networks (p_rewire_ = 1). Figure 5 illustrates the impact of topology on both the fraction of the LSCC and GE over time. Ordered networks degraded rapidly following the crossing of the toxicity threshold (*A*_*th*_ = 25 CL), with LSCC collapsing shortly after *A*_*th*_ ≈ 50 CL. In contrast, small‐world networks exhibited markedly enhanced resilience, maintaining both LSCC integrity and functional efficiency until amyloid levels approached high threshold (*A*_*th*_ *≈ 50 CL)*. Random networks showed intermediate behavior, where the redundancy of long‐range connections delayed structural collapse, but we observed acceleration of the decline in GE due to loss of clustering.

**FIGURE 5:**
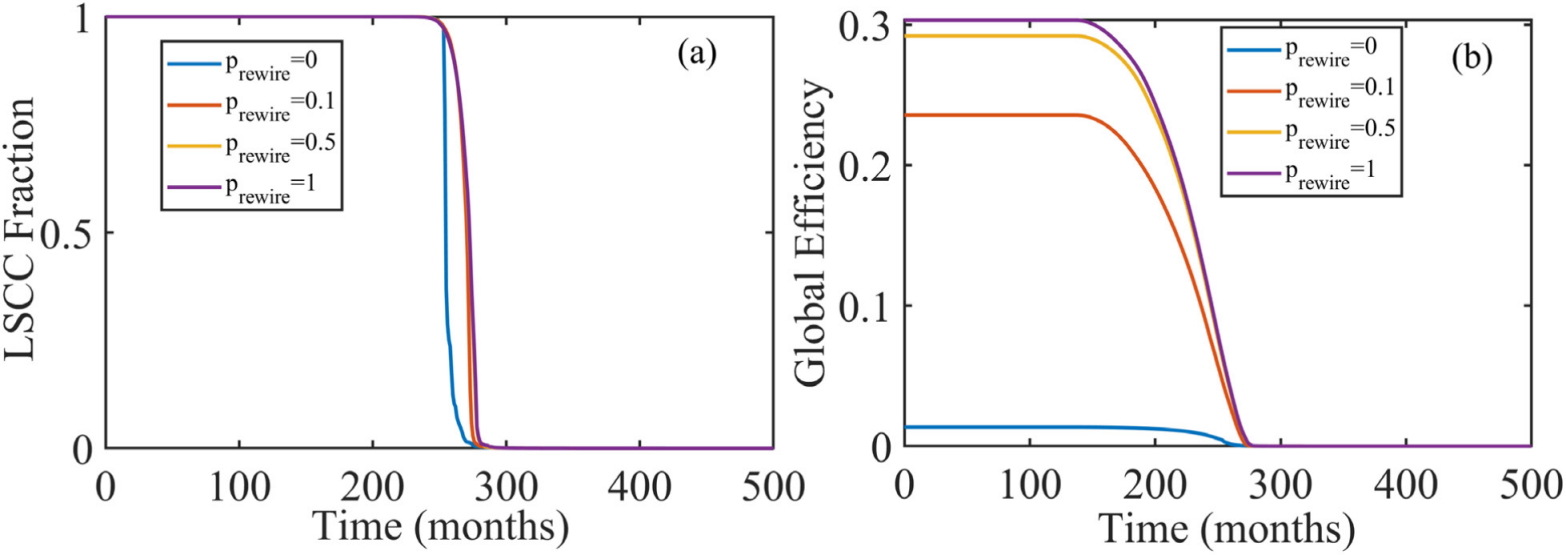
Network topology influences vulnerability to collapse: LSCC fraction (a) and global efficiency (b) over time for different rewiring probabilities p_rewire_. Small‐world topologies (p_rewire_ = 0.1, 0.5) delay structural and functional collapse compared to ordered or random networks (p_rewire_ = 0, 1).

These results highlight a key structural insight: Small‐world topologies optimally balance local clustering and short path lengths, allowing them to absorb localized damage while maintaining global communication (Bassett and Bullmore 2006). The delayed collapse observed in small‐world networks provides a mechanistic explanation for the clinical dissociation between amyloid accumulation and the onset of cognitive symptoms (Sperling et al. 2011), reinforcing the hypothesis that brain architecture has evolved for both efficiency and robustness. Furthermore, the finding that GE consistently declines before LSCC failure across all topologies suggests that GE is a more sensitive and generalizable early‐warning indicator of network failure. This aligns with the concept of critical slowing down as networks approach a tipping point (Scheffer et al. 2012). Collectively, these results emphasize the importance of topological features in determining the timing and severity of ad‐related network degradation and suggest that individual differences in brain connectivity architecture could partly explain the heterogeneity of Alzheimer's progression (Raj et al. 2012).

### 3.5 Eigenvalue Spectrum of the Weighted Adjacency Matrix: A Dynamical Early‐Warning Signal

To directly link the continuous degradation of synaptic weights to the macroscopic dynamical stability of the network, we analyzed the eigenvalue spectrum of the weighted adjacency matrix *W(t)* across disease progression. We define *ρ(t)* as the spectral radius of the weighted adjacency matrix, *ρ(t) = max|λ(t)|*. Because our network is purely excitatory, *W(t)* has nonnegative entries; by the Perron–Frobenius theorem for nonnegative matrices, its leading eigenvalue is guaranteed to be real and nonnegative, so *ρ(t)* coincides with the largest real eigenvalue at every stage of degradation. This quantity is a linear stability criterion around the network's silent (quiescent) state, in the sense used to characterize criticality in excitable networks (Kinouchi and Copelli 2006; Larremore et al. 2011). Two distinct timescales must be distinguished here: the slow, disease‐progression timescale *t* (months), over which amyloid burden degrades *W(t)*; and the fast, spiking timescale *s* (milliseconds), over which neuronal activity evolves on the frozen matrix *W(t)*. Let *p(s)* denote the activation‐probability vector on the fast timescale, whose i‐th entry *p*_*i*_ *(s)* is the probability that neuron *i* is active at fast time step *s*. Under the discrete, linearized branching approximation *p(s + 1) ≈ W(t)·p(s)*, in which each active neuron generates offspring activations with probability proportional to the synaptic weights, small perturbations of the quiescent state grow when *ρ(t) > 1*, decay when *ρ(t) < 1*, and neither grow nor decay at *ρ(t) = 1*. We stress that *p(s)* is an activation probability and is unrelated to the short‐term‐plasticity utilization variable *xij(t)* introduced in Section 2.5.2. *ρ(t)* therefore predicts whether a local perturbation propagates or dies out under this linearized approximation. As amyloid‐driven pruning and synaptic weakening proceed, *ρ(t)* decreases monotonically, crossing 1 at a well‐defined disease stage and continuing toward 0 as the network becomes progressively disconnected.

Figure 6 visualizes the full eigenvalue spectrum in the complex plane at four representative stages. In the healthy stage (*t* = 100 months), the spectrum is broadly distributed in a characteristic crescent shape, with the leading eigenvalue *ρ* ≈ 12.4 extending far beyond the critical stability circle (*|λ|* = 1). As amyloid burden increases, the entire cloud of eigenvalues progressively compresses toward the origin. By the pre‐symptomatic stage (*t* = 200 months), *ρ* drops to 7.7, yet the network remains highly active. Critically, at the midstage transition (*t* = 260 months), the spectrum has compressed dramatically, with *ρ ≈* 1.67 hovering just above the critical threshold. This marks the dynamical tipping point beyond which the network can no longer robustly sustain collective activity. Finally, in the late‐stage silence (*t* = 350 months), the spectrum completely collapses to *ρ* ≈ 0, confirming total loss of dynamical stability. It is important to clarify that *ρ(t)* ≈ 0 at *t* = 350 months does not indicate that every synapse has been pruned. For a weighted directed graph, the spectral radius of the adjacency matrix vanishes if and only if the graph contains no directed cycles (Brualdi 2010; Newman 2018): under a topological ordering of the vertices, the adjacency matrix of an acyclic directed graph is strictly triangular, so all of its eigenvalues are identically zero irrespective of the magnitude of the surviving weights. Thus, *ρ* ≈ 0 quantifies the complete loss of recurrent, reverberating structure rather than the complete absence of connections (the value reported in Figure 6d is zero to numerical precision), rendering the late‐stage network incapable of sustaining self‐maintained activity.

**FIGURE 6:**
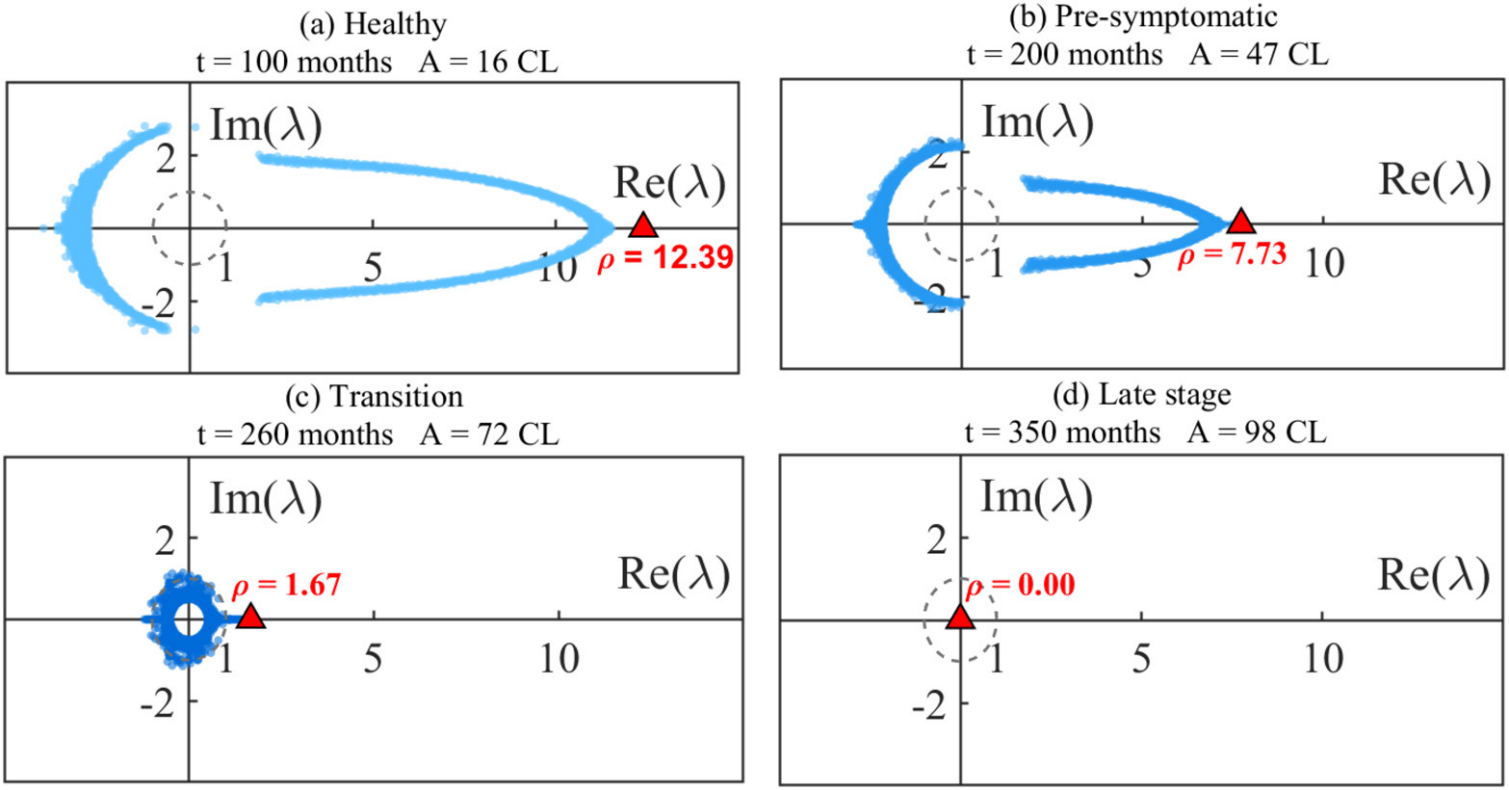
Eigenvalue spectrum of the weighted adjacency matrix *W*(*t*) in the complex plane at four representative disease stages. Each blue point represents an eigenvalue *λ*; the red triangle marks the spectral radius *ρ = max|λ|.* The dashed circle indicates the critical stability threshold (*|λ|* = 1). (a) Healthy stage (*t* = 100 months, *A* = 16 CL): The spectrum exhibits a broad, crescent‐shaped distribution extending to *ρ* = 12.39, reflecting a highly excitable, supercritical network. (b) Pre‐symptomatic stage (*t* = 200 months, *A* = 47 CL): The spectrum contracts but remains robustly supercritical (*ρ* = 7.73). (c) Midstage degradation/transition (*t* = 260 months, *A* = 72 CL): The eigenvalue cloud compresses dramatically toward the origin. The spectral radius (*ρ* = 1.67) hovers just above the critical threshold, marking the dynamical tipping point of the network. (d) Late‐stage silence (*t* = 350 months, *A* = 98 CL): The spectrum collapses to *ρ* ≈ 0.00, indicating a subcritical network incapable of sustaining coordinated activity. Although the macroscopic network has fragmented, a few isolated, weak synaptic connections remain, accounting for the rare, uncorrelated spikes observed in late‐stage spiking simulations.

To quantify the temporal relationship between this dynamical decay and structural collapse, we tracked *ρ(t)* continuously alongside the LSCC fraction (Figure 7). Because *ρ(t)* is sensitive to the continuous weakening of synaptic weights, it begins to decline smoothly well before individual synapses are completely pruned. We observed a significant temporal dissociation: The normalized spectral radius crosses its 50% threshold at *t₁* = 212 months, whereas the structural LSCC fraction reaches its own 50% collapse point much later, at *t*_*2*_ *=* 259 months. This establishes a dynamical early‐warning lead time of approximately 47 months, demonstrating that the spectral radius serves as a highly sensitive, weight‐based precursor to the abrupt, binary topological phase transition captured by the LSCC.

**FIGURE 7:**
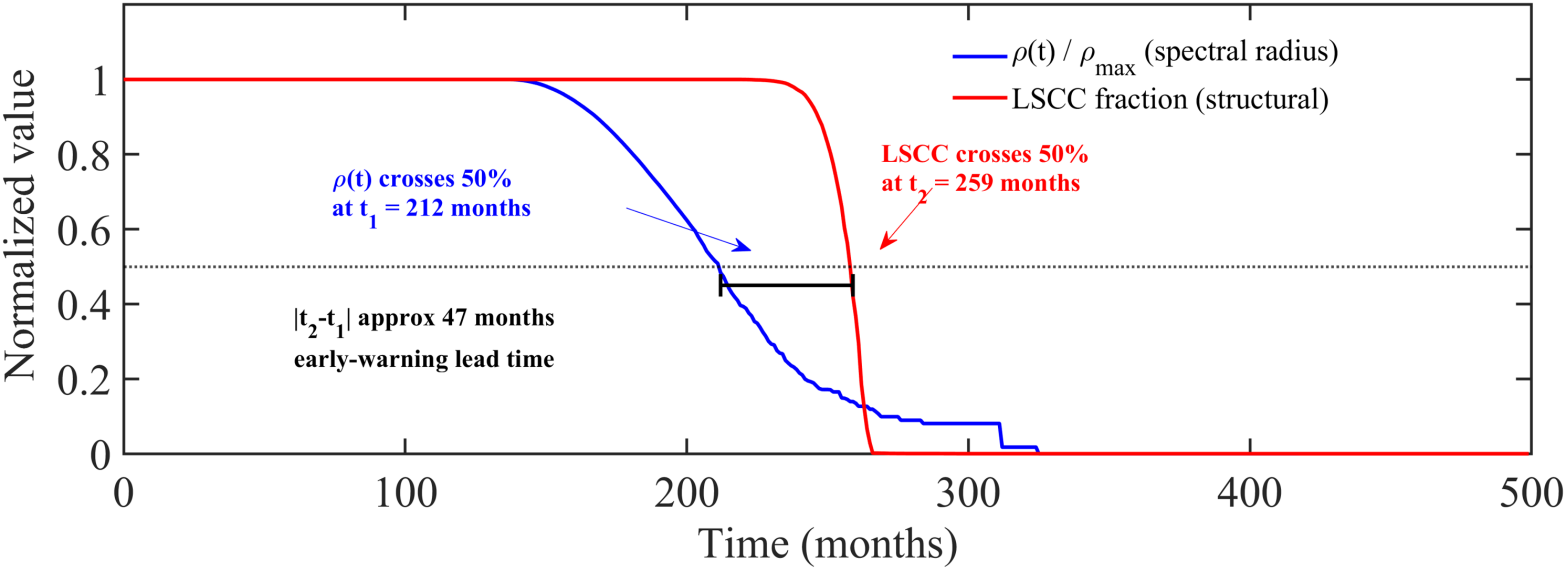
The spectral radius *ρ(t)* acts as a continuous early‐warning signal that anticipates the abrupt structural collapse of the network. The normalized spectral radius (blue) and the largest strongly connected component (LSCC) fraction (red) are plotted over time. Although the structural LSCC remains intact until late in the disease, the spectral radius begins its decline much earlier due to continuous synaptic weakening. The spectral radius crosses its 50% threshold at *t₁* = 212 months, whereas the LSCC crosses its 50% threshold at *t*_*2*_ = 259 months. This reveals a dynamical early‐warning lead time of|*t*_*2*_ *‐t₁*| ≈ 47 months preceding the topological phase transition.

The relation *p(s + 1) ≈ W(t)·p(s)* is invoked to motivate why the spectrum of *W(t)* is dynamically informative, rather than as a formal linearization of our spiking network. A rigorous stability analysis of a network of Izhikevich neurons would require the full state space of membrane potentials and adaptation variables, *y(s + 1) = J(s;t)·y(s)*, where the Jacobian *J(s;t)* combines *W(t)* with intrinsic neuronal dynamics and saltation matrices accounting for the reset discontinuity at each spike (Bizzarri et al. 2013; Aristides and Cerdeira 2024). Although *W(t)* is only one component of *J(s;t)*, it is the only element that varies across disease stages, because all intrinsic neuronal and plasticity parameters are held fixed. Moreover, *ρ(t)* bounds the recurrent gain available to the network: Because the connectivity is purely excitatory and STP can only attenuate the structural weights (Equation 15), the effective connectivity satisfies *Weff(t) ≤ W(t)* entrywise. By the monotonicity of the Perron root for nonnegative matrices, this gives *ρ (Weff(t)) ≤ ρ(t)*. Thus, a value *ρ(t)* < 1 is sufficient for the effective network to be subcritical within the branching approximation. We therefore treat *ρ(t)* as a structural proxy for the capacity of the network to propagate activity, rather than as an exact stability criterion for the spiking dynamics, and we establish its value as an early‐warning signal empirically through its agreement with the simulated firing statistics detailed in Section 4.

## 4 Functional Collapse of Spiking Dynamics

To characterize the functional consequences of amyloid‐induced structural degradation, we simulated spiking dynamics across six key stages of disease progression (100, 200, 260, 275, 300, and 350 months) using an excitatory Izhikevich network augmented with STP. Neurons were simulated on static adjacency matrices extracted at each snapshot, reflecting cumulative synaptic loss due to amyloid burden. Isolated neurons (zero in‐degree) were retained in the network to preserve effective network size but remained quiescent, consistent with biological constraints.

We synthesize multiple dynamical observables that reveal a nuanced, three‐phase collapse. We observe the emergence of functional subpopulations. Dual‐order raster plots sorting neurons by total spike count Panel (a) and Panel (b) in‐degree uncover the progressive formation of three distinct subpopulations (Figure 8). In early stages (*t* ≤ 200 months), activity propagates as wavelike cascades from high‐connectivity neurons to downstream units. By midstage (*t* ≈ 50 months), a tripartite structure dominates: (1) a shrinking core (top 15% by spikes) exhibiting burst‐like firing; (2) a large transient population firing sporadically; and (3) a growing silent fraction (bottom 30%) with no activity. In late stages (*t* ≥ 300 months), the core vanishes, the transient group fragments, and silence prevails (70% inactive).

**FIGURE 8:**
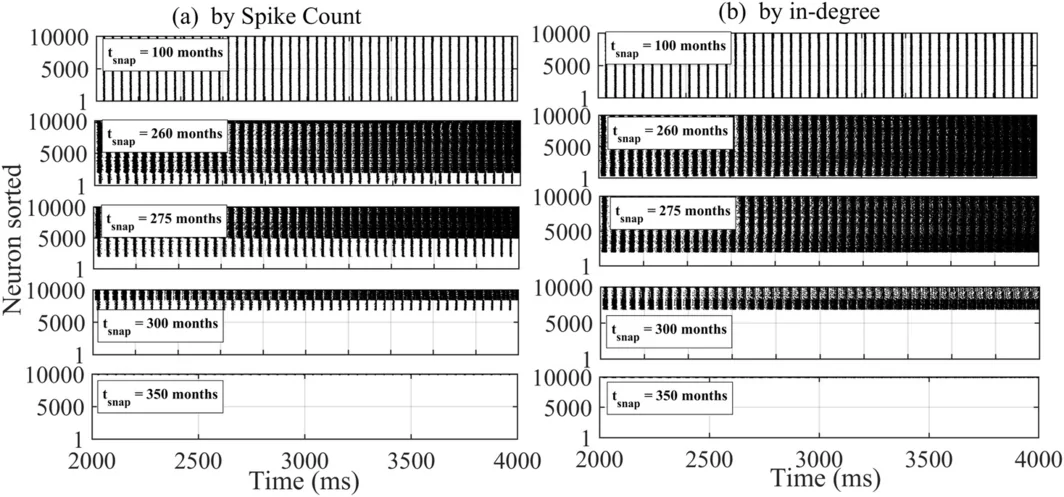
Dual‐order raster plots (sorted by spike count and in‐degree) reveal three emergent functional subpopulations: a hyperactive core, a transient group, and a silent cohort.

The instantaneous population firing rate, $R_{\mathrm{pop}}\left(t\right)=\sum_{i}S_{i}\left(t\right)$, reveals a progressive degradation of collective dynamics (Figure 9). In early stages (*t* ≤ 200 months), *R*_*pop*_ *(t)* exhibits robust, recurrent transients indicative of wavelike propagation and network excitability. By *t* = 275 months, these transients become sparse, irregular, and lower in amplitude, reflecting the fragmentation of functional modules. In late stages (*t* ≥ 300 months), *R*_*pop*_ *(t)* collapses to near‐zero with only rare, uncorrelated spikes, confirming global hypoactivity.

**FIGURE 9:**
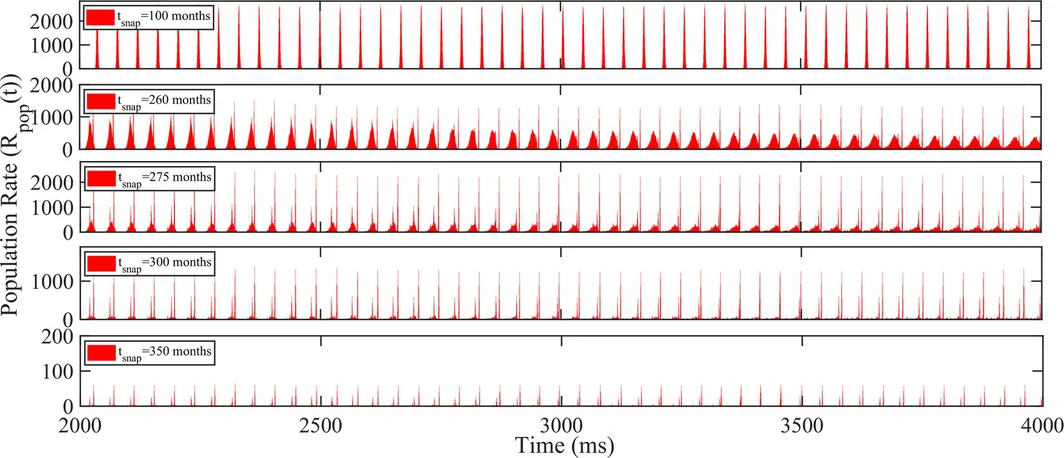
Instantaneous population firing rate showing loss of coordinated transients by midstage and near‐silence in late stages.

Spiking irregularity was quantified using two complementary metrics: the coefficient of variation (CV) of interspike intervals and the Fano factor (spike‐count variance‐to‐mean ratio in 500‐ms sliding windows). As shown in Figure 10 Panel (a), the Fano factor increases dramatically, from ∼0.1 (at *t* = 100 months) to > 4 (at *t* = 350 months), reflecting a transition from network‐coordinated spiking to bursty, stochastic, and asynchronous firing. In stark contrast, the CV remains nearly constant across all disease stages (Figure 10 Panel (b)), indicating that intrinsic neuronal spiking regularity is preserved despite progressive synaptic loss. This dissociation demonstrates that functional collapse arises from the breakdown of network‐level coordination, not from altered single‐neuron dynamics.

**FIGURE 10:**
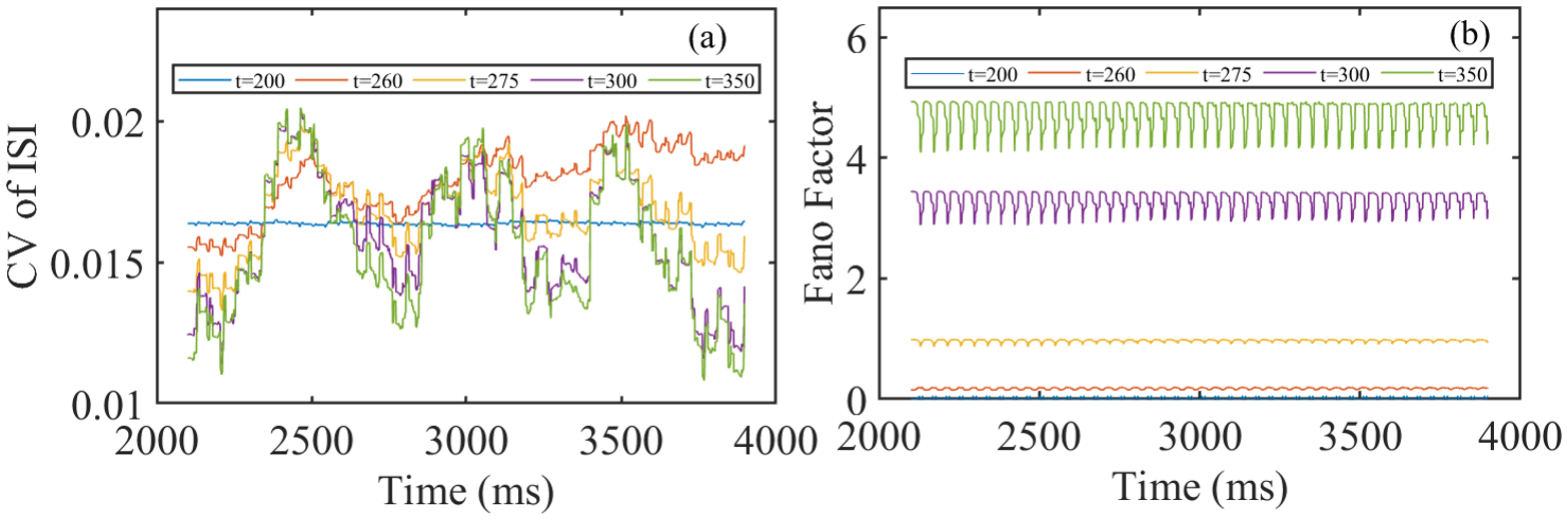
Spiking irregularity during disease progression. (a) The coefficient of variation (CV) of interspike intervals remains approximately constant across all stages, indicating preserved intrinsic spiking regularity. (b) The Fano factor (variance‐to‐mean ratio of spike counts in 500‐ms sliding windows) increases from ∼0.1 to > 4, signaling a shift from coordinated network activity to pathological asynchrony and burstiness. Simulations use an excitatory Izhikevich network with STP at six disease stages (*t* = 100 to 350 months). Parameters: *a =* 0.1, *b* = 0.2, *c* = −65, *d* = 2*, τ*_*facil*_ = 100 ms*, τ*_*rec*_ = 10 ms*, U =* 0.5.

In Figure 11, we observe that as disease progresses, the network undergoes a transition from synchronized, high‐activity states to desynchronized, low‐activity regimes. The mean firing rate of connected neurons remains approximately constant during the early stages of disease progression, exhibits a modest increase around *t* ≈ 200 months and subsequently decreases as structural degradation becomes more pronounced (Figure 11 Panel (c)). Although the increase is relatively small, it indicates that the average firing activity of the connected neuronal population remains largely preserved despite the progressive loss of network connectivity. The error bars, representing the standard deviation of firing rates across connected neurons, remain relatively small throughout the progression, suggesting limited heterogeneity in the firing activity of the connected neuronal population. Although mean firing rate and global synchrony collapse after 150 months, the spectral profile retains its dominant gamma‐band peaks; albeit with reduced power, suggesting that the mechanism for rhythm generation persists even as amplitude fades. This decoupling of frequency stability from power decline may reflect early‐stage compensation followed by late‐stage network failure.

**FIGURE 11:**
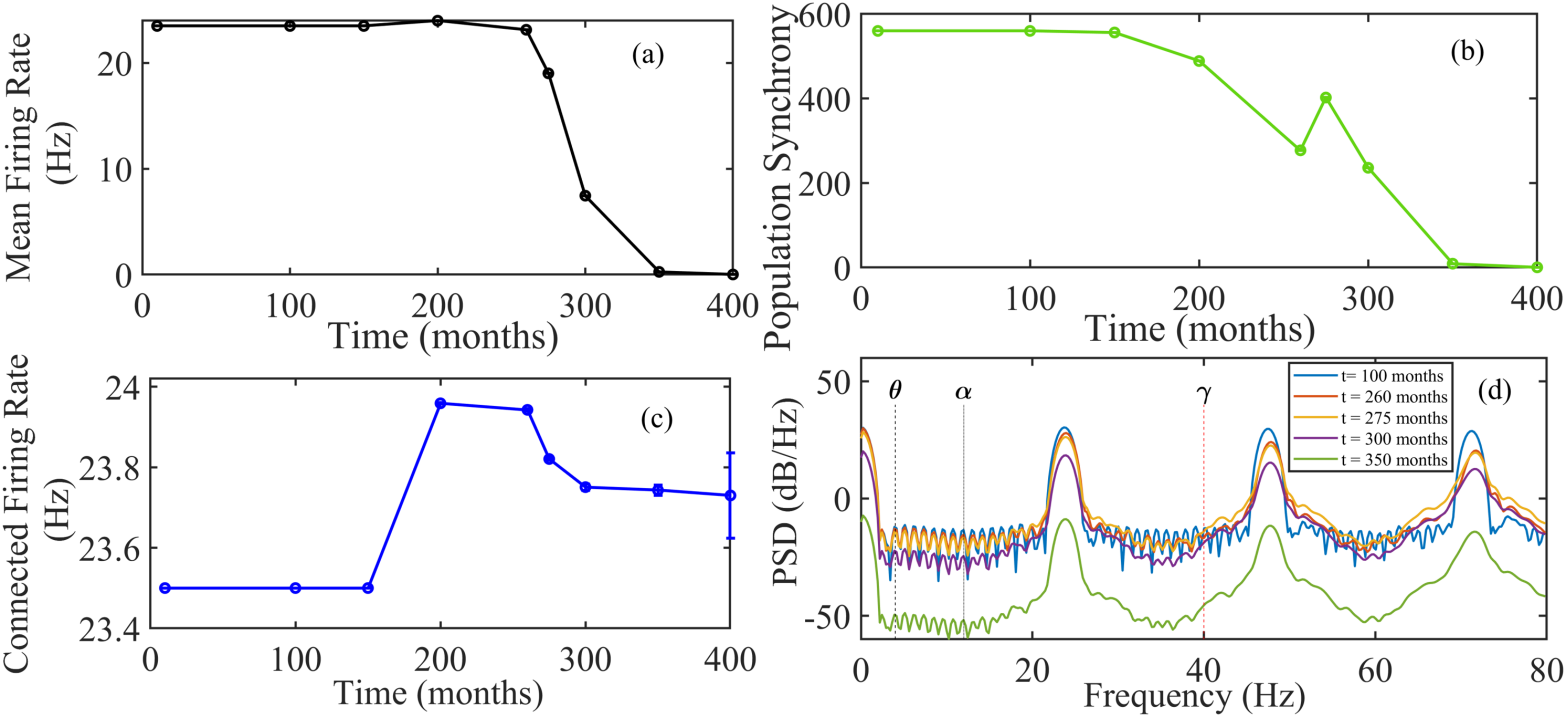
(a) Mean population firing rate declines sharply after *t* = 275 months. (b) Population synchrony, measured as the standard deviation of the instantaneous population firing rate, decreases monotonically with disease progression. (c) The evolution of spiking dynamics during amyloid‐induced network degradation. (c) Mean firing rate of connected neurons. Error bars represent the standard deviation of firing rates across connected neurons at each disease stage. (d) Power spectral density (PSD) reveals persistent gamma‐band oscillations (25–75 Hz) across all stages, but power diminishes significantly at late stages (*t* = 350 months), indicating loss of high‐frequency synchrony. Theta (*θ,*4 Hz), alpha (*α,*12 Hz), and gamma (*γ,*40 Hz) bands are marked for reference. Parameters: *a* = 0.1*, b* = 0.2*, c* = −65*, d* = 0.2*, τ*_*facil*_ = 100 ms*, τ*_*rec*_ *=* 10 ms*, U* = 0.5.

Collectively, these results refine the original two‐phase model into a more biologically grounded trajectory:

- A decoupled propagation phase (*t* ≤ 250 months): intact structure but disordered function, with emergent subpopulations, minimal hub dominance, and intact population transients.
- A reorganization phase (250 ≤ *t* ≤ 300 months): rising variability, spectral slowing, fragmented population bursts, and partial restoration of structure–function alignment as resilient hubs sustain residual activity
- A collapsed silence phase (*t* ≈ 300 months): asynchrony, near‐zero population rate, artifactual rank correlation, and loss of all collective dynamics.

This progression aligns with multimodal clinical data particularly the paradoxical coexistence of focal hyperactivity and widespread hypoactivity in early Alzheimer's disease and establishes structured raster analysis, rank dynamics, and population rate decomposition as sensitive, multiscale biomarkers of network degradation.

Additionally, the inclusion of STP in the model, particularly synaptic facilitation, may contribute to the maintenance of synchrony in early stages. STP dynamically enhances synaptic transmission during repetitive activity, thereby reinforcing transient coordination across neurons even as structural integrity degrades. Although not isolated in this study, its role likely complements topological resilience in delaying functional disintegration (Zucker and Regehr 2002).

## 5 Discussion

Our findings highlight the multifactorial nature of Alzheimer's disease progression, in which molecular, structural, and dynamical elements interact across scales. Using a computational model that integrates amyloid accumulation, synaptic pruning, and biologically realistic neuronal dynamics, we revealed key mechanisms governing the resilience and collapse of brain networks in Alzheimer's disease.

First, our results reinforce the critical role of network topology. Small‐world architectures, which balance high clustering with short path lengths, confer significant resilience against amyloid‐induced degradation. These networks exhibit delayed collapse of both structural (LSCC) and functional (GE) markers compared to ordered or random configurations (Figure 5), supporting the hypothesis that small‐world connectivity has evolved to optimize robustness (Bassett and Bullmore 2006; Mišić et al. 2015). Notably, GE declines (Figure 1 and Figure 2) prior to LSCC collapse across all topologies, suggesting that functional metrics may serve as early‐warning indicators of network failure (Scheffer et al. 2012).

Second, we observed a clear dissociation between amyloid deposition and the timing of network collapse. The functional breakdown lags behind the molecular pathology (Figure 1), mirroring clinical observations where amyloid accumulation precedes cognitive decline by a decade or more (Sperling et al. 2011; Villemagne et al. 2013). The inclusion of synaptic time constants (*τ*_*s*_) in our model further refines this timeline: Longer *τ*_*s*_ values extend the network's compensatory phase, delaying collapse and increasing the critical amyloid burden required to trigger failure (Figure 3 and Figure 4). This aligns with the cognitive reserve hypothesis (Stern 2012), suggesting that individuals with slower synaptic degradation may sustain function longer despite equivalent amyloid load.

Third, we identified the spectral radius *ρ(t)* as a highly sensitive, weight‐based early‐warning indicator that anticipates structural collapse by approximately 47 months (Figure 7). Unlike GE, which depends on binary connectivity, the spectral radius continuously tracks progressive synaptic weakening through the eigenvalue spectrum of the weighted adjacency matrix. We observed that *ρ(t)* begins its decline immediately after amyloid crosses the toxicity threshold (*A*_*th*_ = 25 CL), whereas the LSCC remains intact until much later. This temporal dissociation reveals that dynamical destabilization precedes topological fragmentation, establishing *ρ(t)* as a more sensitive indicator of early network dysfunction than purely structural metrics.

The complex eigenvalue spectrum (Figure 6) reflects the directed, asymmetric nature of synaptic connectivity rather than intrinsic oscillations. The characteristic crescent‐shaped distribution is a mathematical signature of directed small‐world networks, where only the spectral radius *ρ(t)*, which is the maximum modulus eigenvalue, has direct dynamical interpretation: It determines whether linearized perturbations grow or decay. The imaginary components encode directional asymmetry but are not biologically interpretable as oscillatory modes because neuronal firing is governed by nonlinear Izhikevich dynamics rather than the linear spectrum of *W* itself. This distinction differentiates our directed network framework from studies using undirected graphs, where eigenvalues are necessarily real and lack this geometric richness. From a clinical perspective, these findings suggest that functional biomarkers sensitive to synaptic weight changes may detect ad‐related dysfunction years before structural disconnection becomes evident on diffusion tensor imaging. Recent work has demonstrated that statistical markers can successfully estimate the closeness of a neuronal population to a critical point (Sooter et al. 2025; Chopra et al. 2026), which could serve as a viable experimental proxy for the spectral radius in future empirical studies.

Our spiking‐network simulations reveal how amyloid‐driven synaptic loss reshapes neuronal population dynamics across disease progression. By analyzing activity across six stages from preclinical (100 months) to advanced dementia (*t* = 350 months), we identify a three‐phase functional trajectory that refines the traditional view of Alzheimer's disease as a simple hypoactivity syndrome. At the single‐neuron resolution, dual‐order raster plots (Figure 8) uncover the progressive emergence of three functionally distinct subpopulations: (1) a hyperactive core (top 15% by spike count) exhibiting burst‐like firing; (2) a large transient group with sporadic activity; and (3) a silent cohort (> 70% by late stage) with no spiking output. This tripartite organization mirrors clinical electrophysiological data showing coexisting focal hyperactivity and widespread hypoactivity in early Alzheimer's disease (Palop and Mucke 2016). The alignment between structural connectivity (in‐degree) and functional output (spike count) degrades over time, reflecting a progressive decoupling of structure and function. At the macroscopic network level, the instantaneous population firing rate (Figure 11) demonstrates a clear transition from coordinated, wavelike transients in early stages to fragmented, sparse bursts by midstage, and finally to near‐complete silence in late stages. This confirms that network‐level coordination not just mean firing rate is the first casualty of synaptic degradation. Providing critical mechanistic insight, the dissociation between Fano factor and coefficient of variation (Figure 10) provides critical mechanistic insight. The Fano factor, which measures spike‐count variability across time, increases and fold (from ~0.1 to 4), signaling a shift from reliable, network‐driven spiking to pathological burstiness and asynchrony. In stark contrast, the coefficient of variation of interspike intervals remains stable, indicating that intrinsic neuronal spiking regularity is preserved throughout disease progression. This confirms that functional collapse arises from loss of network‐level coordination, not altered single‐neuron dynamics, a key distinction for biomarker development and therapeutic targeting (Churchland et al. 2010; Palop and Mucke 2016). Ultimately, metrics of population synchrony and mean firing rate (Figure 11) quantify the collapse of collective dynamics. Both decline sharply after the critical accumulated dose is reached (corresponding to ~75 CL in our baseline simulations), coinciding with the disappearance of the hyperactive core and the fragmentation of the transient population. Together, these results establish that Alzheimer's disease‐related network failure is not uniform but instead involves dynamic reorganization into functionally segregated subpopulations, followed by progressive disintegration of all coordinated activity. Interestingly, the decline in neuronal activity occurs more gradually than the corresponding structural transition, suggesting that nonlinear neuronal dynamics and short‐term synaptic facilitation can temporarily sustain activity despite progressive network degradation. A systematic finite‐size scaling analysis of these spiking observables would therefore be valuable to determine whether the transition sharpens with increasing network size, similarly to the finite‐size behavior observed for the LSCC. Such an investigation is beyond the scope of the present work and will be addressed in future studies.

These findings highlight the value of multiscale spiking, particularly structured raster analysis, Fano factor, and population synchrony, as sensitive biomarkers of early network dysfunction. They also reinforce the view of Alzheimer's disease as a systems‐level cascade, where synaptic loss disrupts emergent population dynamics long before global silence sets in.

The selection of network architecture, neuronal dynamics, and synaptic plasticity rules was guided by biological relevance, computational tractability, and the specific multiscale objectives of this study. At the network level, we adopted a Watts–Strogatz small‐world topology because it accurately captures the high local clustering and short path lengths characteristic of mammalian cortical and hippocampal microcircuits (Stam et al. 2007; Markram et al. 2015). This choice is particularly pertinent for Alzheimer's disease modeling, as early pathology preferentially targets the hippocampus and entorhinal cortex, regions that exhibit pronounced small‐world organization that systematically degrades as amyloid burden increases (Sperling et al. 2011; Markram et al. 2015).

Regarding neuronal dynamics, we employed the Izhikevich spiking neuron model rather than traditional rate‐based frameworks (e.g., Wilson–Cowan, mean‐field, or population firing‐rate models). Rate‐based models inherently average out precise spike timing, temporal correlations, and burst dynamics, which are critical for capturing the transient compensatory activity and pathological asynchrony observed in early Alzheimer's disease (Palop and Mucke 2016). For our excitatory population, we utilized parameters corresponding to the fast‐recovery spiking regime (*a* = 0.1*, b* = 0.2*, c* = −65*, d* = 2) (Izhikevich 2003). In the context of our purely excitatory network, this parameter set is computationally necessary to ensure rapid recovery of the membrane potential, sustain robust baseline firing rates, and support the emergence of collective dynamical states (including synchronization and transient oscillatory patterns). This provides a robust functional baseline from which amyloid‐induced alterations and network collapse can be accurately quantified.

At the synaptic level, we incorporated short‐term synaptic plasticity to model millisecond‐to‐second modulation of synaptic efficacy during repetitive firing. We deliberately excluded long‐term plasticity rules (spiking‐time–dependent plasticity, long‐term potentiation/depression), which operate on timescales of hours to days. Including such mechanisms would overlap with the structural degradation timescale (months to years), violating the quasistatic approximation required to isolate the impact of cumulative connectivity loss on instantaneous spiking dynamics. STP, particularly facilitation, plays a specific functional role in our model: By dynamically enhancing synaptic efficacy during repeated activation, it enables the network to temporarily maintain coordinated spiking despite gradual structural decay, thereby contributing to the transient synchrony preservation observed during early disease stages (Zucker and Regehr 2002).

Finally, we acknowledge that alternative modeling choices exist. Scale‐free topologies, random graphs, or biophysically detailed Hodgkin–Huxley neurons could be substituted; however, such alternatives would primarily shift the exact numerical thresholds or computational demands without altering the core qualitative findings. Our simulations across varying rewiring probabilities (Section 3.4) already demonstrate that while topology modulates the timing of collapse, the biphasic degradation trajectory, the early decline of GE, and the emergence of functional subpopulations remain robust. Thus, although parsimonious by design, our framework successfully isolates the fundamental interplay between amyloid‐driven structural decay and emergent network dynamics.

Although our model omits inhibitory neurons, our results align with broader principles observed in pathological network dynamics, namely, that the interplay between structural connectivity and activity propagation determines whether a perturbation dissipates or cascades. For example, in epilepsy, seizure‐like inputs can either be contained or amplified depending on inhibitory control and network architecture (Depannemaecker et al. 2022). In contrast, in amyloid‐driven Alzheimer's disease models, the gradual erosion of excitatory connectivity leads not to runaway excitation but to a failure of propagation and eventual silencing, illustrating two divergent failure modes of brain network resilience.

In sum, our work emphasizes that Alzheimer's disease cannot be fully understood without considering the interplay between intrinsic neuronal properties, synaptic dynamics, and global network topology. These insights support the use of multiscale computational models to identify early biomarkers, understand individual variability, and predict the impact of potential interventions. Future work should focus on integrating patient‐specific connectomes and cell‐type heterogeneity to move toward personalized modeling and therapeutic targeting.

## 6 Conclusion

This study presents a multiscale computational framework that bridges molecular pathology and network‐level dysfunction in Alzheimer's disease. By modeling amyloid accumulation, synaptic degradation, STP, and spiking neuron dynamics on biologically inspired small‐world networks, we identified critical mechanisms governing the resilience and collapse of brain connectivity.

Theoretically, our findings demonstrate that small‐world topologies confer superior resistance to amyloid‐induced failure, and that GE serves as a robust early‐warning indicator of collapse. Synaptic decay time constants (*τ*_*s*_) modulate the delay between amyloid accumulation and functional breakdown, offering a biophysical explanation for the clinical dissociation between pathology and symptoms. Our spiking‐network simulations demonstrate that amyloid‐induced synaptic loss drives a progressive and structured disintegration of population dynamics, culminating in global functional silence. Across six disease stages, we observe a three‐phase trajectory: (i) an early propagation phase with wavelike, hub‐driven activity; (ii) a midstage reorganization phase characterized by the emergence of three functional subpopulations, a hyperactive core, a transient group, and a growing silent cohort alongside rising population variability; and (iii) a late collapsed phase dominated by asynchrony, near‐zero firing, and loss of all collective dynamics.

Critically, the Fano factor increases more than 40fold, whereas the coefficient of variation of interspike intervals remains stable, revealing a dissociation between population level desynchronization and preserved single‐neuron spiking regularity. This confirms that network collapse arises from loss of connectivity not intrinsic neuronal dysfunction and establishes Fano factor, structured raster analysis, and population synchrony as sensitive, early biomarkers of network failure.

These findings provide a dynamic counterpart to structural models of Alzheimer's progression and emphasize that functional degradation follows a nonuniform, hierarchical pattern long before global silence sets in. The framework offers a foundation for interpreting electrophysiological biomarkers in early Alzheimer's disease and for evaluating interventions aimed at preserving network coordination.

Clinically, our results suggest that dynamic functional biomarkers, such as declining synchrony, rising entropy, and GE fluctuations, may be more sensitive indicators of early dysfunction than static structural metrics. The model identifies the 25 CL threshold as the tipping point beyond which endogenous repair becomes ineffective, reinforcing the importance of early intervention. Delaying synaptic weakening or enhancing intrinsic plasticity could, in principle, extend network resilience by 50–100 months, depending on synaptic kinetics.

Although our model integrates key biological and network‐level features of Alzheimer's progression, several limitations must be noted. First, the model focuses on amyloid‐beta pathology and does not explicitly include tau protein dynamics, neuroinflammation, or glial interactions, which are known to influence disease progression. Second, we use homogeneous neurons and generic network topologies rather than patient‐specific connectomes or cell‐type distributions. Third, although repair mechanisms and STP are included, other forms of plasticity such as long‐term potentiation/depression are not modeled. Future work should extend this framework to incorporate multimodal pathology, heterogeneous connectome data, and personalized modeling to improve clinical translation.

Future work should focus on patient‐specific modeling, incorporating individual connectomes, diverse neuron types, and copathologies such as tau propagation or neuroinflammation. The current framework provides a foundation for testing personalized interventions and for developing computational biomarkers to support early diagnosis and prognosis in Alzheimer's disease. Ultimately, this approach offers both mechanistic insight and translational potential in the study of neurodegenerative network collapse.

## Author Contributions

**Ediline L. F. Nguessap:** methodology, validation, visualization, writing – review and editing, investigation, conceptualization, writing – original draft, formal analysis, data curation, software. **Damien Depannemaecker:** conceptualization, methodology, investigation, formal analysis, supervision, writing – review and editing, validation, visualization. **Fernando F. Ferreira:** conceptualization, investigation, funding acquisition, writing – original draft, writing – review and editing, methodology, formal analysis, project administration, supervision, validation.

## Ethics Statement

The authors have nothing to report.

## Conflicts of Interest

The authors declare no conflicts of interest.

## Data Availability

No empirical data generated. All code for model implementation, simulations, and figures is publicly available for reproducibility at: https://github.com/ediline000/amyloid‐network‐collapse.git.

## References

[ejn70713-bib-0001] Arendt, T. 2009. “Synaptic Degeneration in Alzheimer's Disease.” Acta Neuropathologica 118, no. 1: 167–179. 10.1007/s00401-009-0536-x 19390859

[ejn70713-bib-0002] Aristides, R. P. , and H. A. Cerdeira . 2024. “Master Stability Functions of Networks of Izhikevich Neurons.” Physical Review E 109, no. 4: 044213. 10.1103/PhysRevE.109.044213 38755844

[ejn70713-bib-0003] Bassett, D. S. , and E. D. Bullmore . 2006. “Small‐World Brain Networks.” Neuroscientist: A Review Journal Bringing Neurobiology, Neurology and Psychiatry 12, no. 6: 512–523. 10.1177/1073858406293182 17079517

[ejn70713-bib-0004] Bizzarri, F. , A. Brambilla , and G. S. Gajani . 2013. “Lyapunov Exponents Computation for Hybrid Neurons.” Journal of Computational Neuroscience 35, no. 2: 201–212. 10.1007/s10827-013-0448-6 23463130

[ejn70713-bib-0005] Brualdi, R. A. 2010. “Spectra of Digraphs.” Linear Algebra and Its Applications 432, no. 9: 2161–2196.

[ejn70713-bib-0006] Burgold, S. , S. Filser , M. M. Dorostkar , B. Schmidt , and J. Herms . 2014. “In Vivo Imaging Reveals Sigmoidal Growth Kinetic of β‐Amyloid Plaques.” Acta Neuropathologica Communications 2, no. 1: 30. 10.1186/2051-5960-2-30 PMC4050984 24678659

[ejn70713-bib-0007] Chia, R. S. L. , K. Minta , L.‐Y. Wu , et al. 2024. “Serum Brevican as a Biomarker of Cerebrovascular Disease in an Elderly Cognitively Impaired Cohort.” Biomolecules 14, no. 1: 75. 10.3390/biom14010075 PMC10813026 38254675

[ejn70713-bib-0008] Chopra, R. , J. Zhong , E. Miller , et al. 2026. “A Single Dynamical Property Can Account for the Capacity to Learn, From Artificial Networks to the Mammalian Brain.” bioRxiv: 2026‐07.09.737603. 10.64898/2026.07.09.737603.

[ejn70713-bib-0009] Churchland, A. K. , B. M. Yu , J. P. Cunningham , et al. 2010. “Stimulus Onset Quenches Neural Variability: A Widespread Cortical Phenomenon.” Nature Neuroscience 13, no. 3: 369–378. 10.1038/nn.2501 PMC2828350 20173745

[ejn70713-bib-0010] de Haan, W. , E. C. W. van Straaten , A. A. Gouw , and C. J. Stam . 2017. “Altering Neuronal Excitability to Preserve Network Connectivity in a Computational Model of Alzheimer's Disease.” PLoS Computational Biology 13, no. 9: e1005707. 10.1371/journal.pcbi.1005707 PMC5627940 28938009

[ejn70713-bib-0011] Dehghani, N. , A. Peyrache , L. A. Debasco , et al. 2016. “Dynamic Balance of Excitation and Inhibition in Human and Monkey Neocortex.” Scientific Reports 6: 23176. 10.1038/srep23176 PMC4793223 26980663

[ejn70713-bib-0012] DeKosky, S. T. , and S. W. Scheff . 1990. “Synapse Loss in Frontal Cortex Biopsies in Alzheimer's Disease: Correlation With Cognitive Severity.” Annals of Neurology 27, no. 5: 457–464. 10.1002/ana.410270502 2360787

[ejn70713-bib-0013] Depannemaecker, D. , M. Carlu , J. Bouté , and A. Destexhe . 2022. “A Model for the Propagation of Seizure Activity in Normal Brain Tissue.” eNeuro 9, no. 6: ENEURO.0234–21.2022. 10.1523/ENEURO.0234-21.2022 PMC9721309 36323513

[ejn70713-bib-0014] Gu, Y. , Y. Lin , L. Huang , and Z. Liu . 2021. “Multilayer Network Analysis of Amyloid‐Beta Propagation in Alzheimer's Disease.” NeuroImage 244: 118598.

[ejn70713-bib-0015] Izhikevich, E. M. 2003. “Simple Model of Spiking Neurons.” IEEE Transactions on Neural Networks 14, no. 6: 1569–1572. 10.1109/TNN.2003.820440 18244602

[ejn70713-bib-0016] Jack, C. R., Jr. , D. A. Bennett , K. Blennow , et al. 2018. “NIA‐AA Research Framework: Toward a Biological Definition of Alzheimer's Disease.” Alzheimer's & Dementia 14, no. 4: 535–562. 10.1016/j.jalz.2018.02.018 PMC5958625 29653606

[ejn70713-bib-0017] Jack, C. R., Jr. , D. S. Knopman , W. J. Jagust , et al. 2013. “Tracking Pathophysiological Processes in Alzheimer's Disease: An Updated Hypothetical Model of Dynamic Biomarkers.” Lancet Neurology 12, no. 2: 207–216. 10.1016/S1474-4422(12)70291-0 PMC3622225 23332364

[ejn70713-bib-0018] Jagust, W. J. , and S. M. Landau . 2021. “Temporal Dynamics of β‐Amyloid Accumulation in Aging and Alzheimer Disease.” Neurology 96, no. 9: e1347–e1357. 10.1212/WNL.0000000000011524 PMC8055327 33408147

[ejn70713-bib-0019] Jones, D. T. , D. S. Knopman , J. L. Gunter , et al. 2016. “Cascading Network Failure Across the Alzheimer's Disease Spectrum.” Brain 139, no. 2: 547–562. 10.1093/brain/awv338 PMC4805086 26586695

[ejn70713-bib-0020] Kashyap, G. , D. Bapat , D. Das , et al. 2019. “Synapse Loss and Progress of Alzheimer's Disease—A Network Model.” Scientific Reports 9, no. 1: 6555. 10.1038/s41598-019-43076-y PMC6484103 31024073

[ejn70713-bib-0021] Kinouchi, O. , and M. Copelli . 2006. “Optimal Dynamical Range of Excitable Networks at Criticality.” Nature Physics 2, no. 5: 348–351.

[ejn70713-bib-0022] Klunk, W. E. , R. A. Koeppe , J. C. Price , et al. 2022. “The Centiloid Project.” Alzheimer's & Dementia 18, no. 1: 1–15. 10.1016/j.jalz.2014.07.003 PMC4300247 25443857

[ejn70713-bib-0023] Larremore, D. B. , W. L. Shew , and J. G. Restrepo . 2011. “Predicting Criticality and Dynamic Range in Complex Networks: Effects of Topology.” Physical Review Letters 106, no. 5: 058101. 10.1103/PhysRevLett.106.058101 21405438

[ejn70713-bib-0024] Markram, H. , J. Lübke , M. Frotscher , and B. Sakmann . 1998. “Regulation of Synaptic Efficacy by Coincidence of Postsynaptic APs and EPSPs.” Science 275, no. 5297: 213–215. 10.1126/science.275.5297.213 8985014

[ejn70713-bib-0025] Markram, H. , E. Muller , S. Ramaswamy , et al. 2015. “Reconstruction and Simulation of Neocortical Microcircuitry.” Cell 163, no. 2: 456–492. 10.1016/j.cell.2015.09.029 26451489

[ejn70713-bib-0026] Mišić, B. , O. Sporns , and A. R. McIntosh . 2015. “Cooperative and Competitive Spreading Dynamics on the Human Connectome.” Neuron 86, no. 6: 1518–1529. 10.1016/j.neuron.2015.05.035 26087168

[ejn70713-bib-0027] Newman, M. E. J. 2018. Networks. 2nd ed. Oxford University Press.

[ejn70713-bib-0028] Palop, J. J. , and L. Mucke . 2016. “Network Abnormalities and Interneuron Dysfunction in Alzheimer Disease.” Nature Reviews Neuroscience 17, no. 12: 777–792. 10.1038/nrn.2016.141 PMC8162106 27829687

[ejn70713-bib-0029] Raj, A. , A. Kuceyeski , and M. Weiner . 2012. “A Network Diffusion Model of Disease Progression in Dementia.” Neuron 73, no. 6: 1204–1215. 10.1016/j.neuron.2011.12.040 PMC3623298 22445347

[ejn70713-bib-0030] Scheffer, M. , S. R. Carpenter , T. M. Lenton , et al. 2012. “Anticipating Critical Transitions.” Science 338, no. 6105: 344–348. 10.1126/science.1225244 23087241

[ejn70713-bib-0031] Selkoe, D. J. , and J. Hardy . 2016. “The Amyloid Hypothesis of Alzheimer's Disease at 25 Years.” EMBO Molecular Medicine 8, no. 6: 595–608. 10.15252/emmm.201606210 PMC4888851 27025652

[ejn70713-bib-0032] Sooter, J. S. , A. J. Fontenele , A. K. Barreiro , C. Ly , K. B. Hengen , and W. L. Shew . 2025. “Defining and Measuring Proximity to Criticality.” bioRxiv: 2025.08.03.668332. 10.1101/2025.08.03.668332.

[ejn70713-bib-0033] Sperling, R. A. , P. S. Aisen , L. A. Beckett , et al. 2011. “Toward Defining the Preclinical Stages of Alzheimer's Disease: Recommendations From the National Institute on Aging‐Alzheimer's Association Workgroups on Diagnostic Guidelines for Alzheimer's Disease.” Alzheimer's & Dementia 7, no. 3: 280–292. 10.1016/j.jalz.2011.03.003 PMC3220946 21514248

[ejn70713-bib-0034] Stam, C. J. , B. F. Jones , G. Nolte , M. Breakspear , and P. Scheltens . 2007. “Small‐World Networks and Functional Connectivity in Alzheimer's Disease.” Cerebral Cortex 17, no. 1: 92–99. 10.1093/cercor/bhj127 16452642

[ejn70713-bib-0035] Stern, Y. 2012. “Cognitive Reserve in Ageing and Alzheimer's Disease.” Lancet Neurology 11, no. 11: 1006–1012. 10.1016/S1474-4422(12)70191-6 PMC3507991 23079557

[ejn70713-bib-0036] Tampellini, D. 2015. “Synaptic Activity and Alzheimer's Disease: A Critical Update.” Frontiers in Neuroscience 9: 423. 10.3389/fnins.2015.00423 PMC4631827 26582973

[ejn70713-bib-0037] Terry, R. D. , E. Masliah , D. P. Salmon , et al. 1991. “Physical Basis of Cognitive Alterations in Alzheimer's Disease: Synapse Loss Is the Major Correlate of Cognitive Impairment.” Annals of Neurology 30, no. 4: 572–580. 10.1002/ana.410300410 1789684

[ejn70713-bib-0038] Villemagne, V. L. , S. Burnham , P. Bourgeat , et al. 2013. “Amyloid β Deposition, Neurodegeneration, and Cognitive Decline in Sporadic Alzheimer's Disease: A Prospective Cohort Study.” Lancet Neurology 12, no. 4: 357–367. 10.1016/S1474-4422(13)70044-9 23477989

[ejn70713-bib-0039] Vogels, T. P. , R. C. Froemke , N. Doyon , et al. 2011. “Inhibitory Plasticity Balances Excitation and Inhibition.” Science 334, no. 6062: 1569–1573. 10.1126/science.1211095 22075724

[ejn70713-bib-0040] Yuan, P. , C. Condelo , Z. Hou , et al. 2023. “Microglial Clearance Mechanisms in Alzheimer's Disease.” Nature Neuroscience 26: 406–418.

[ejn70713-bib-0041] Zhang, X. , L. Wang , K. Chen , et al. 2023. “Compensatory Axonal Sprouting in Early Alzheimer's Disease: A Longitudinal PET Study.” Science Translational Medicine 15, no. 712: eabq3871.

[ejn70713-bib-0042] Zucker, R. S. , and W. G. Regehr . 2002. “Short‐Term Synaptic Plasticity.” Annual Review of Physiology 64, no. 1: 355–405. 10.1146/annurev.physiol.64.092501.114547 11826273

